# Correction: *In silico* and *in vitro* studies on the anti-cancer activity of andrographolide targeting survivin in human breast cancer stem cells

**DOI:** 10.1371/journal.pone.0247694

**Published:** 2021-02-23

**Authors:** Septelia Inawati Wanandi, Agus Limanto, Elvira Yunita, Resda Akhra Syahrani, Melva Louisa, Agung Eru Wibowo, Sekar Arumsari

The images for Fig 5A and 5B are incorrectly switched. The image that appears as Fig 5A should be Fig 5B, and the image that appears as Fig 5B should be Fig 5A. The figure captions appear in the correct order.

**Fig 5:**
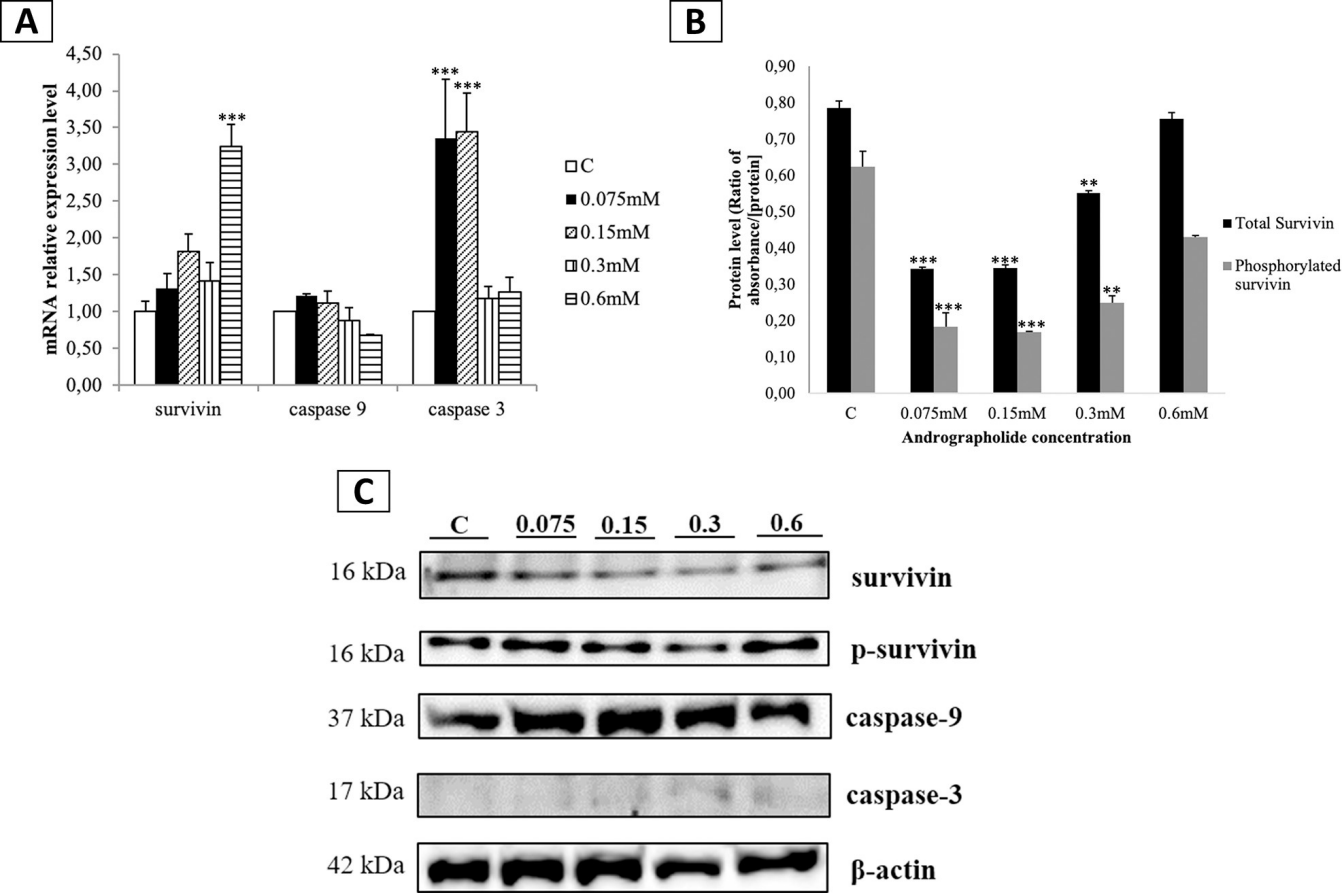
Effect of andrographolide on the expression levels of survivin, caspase-9, and caspase-3 in BCSCs. (A) mRNA expression levels of survivin, caspase-9, and caspase-3 in BCSCs treated with various concentrations of andrographolide; (B) Protein levels of total survivin and Thr34-phosphorylated survivin in BCSCs treated with various concentrations of andrographolide analyzed using ELISA; (C) Immunoassay results of survivin, Thr34-phosphorylated survivin, active caspase-9, and active caspase-3; C: control cells treated with DMSO 0.01% (vehicle). Data (A) and (B) were shown as the mean ± SD from three independent experiments. One-way ANOVA followed by Tuckey’s multiple comparison tests were used to determine mean differences between groups. Statistical significance is shown in the figure as follows: **p<0.01 and ***p<0.001 compared to control.

In Fig 5A and 5B, the signs for statistical significance (** and ***) were missing. Please see the correct figures here.

In Fig 7, the word “survivin” in the upper right blue box should appear as “p-survivin”. Please see the correct figure here.

**Fig 7:**
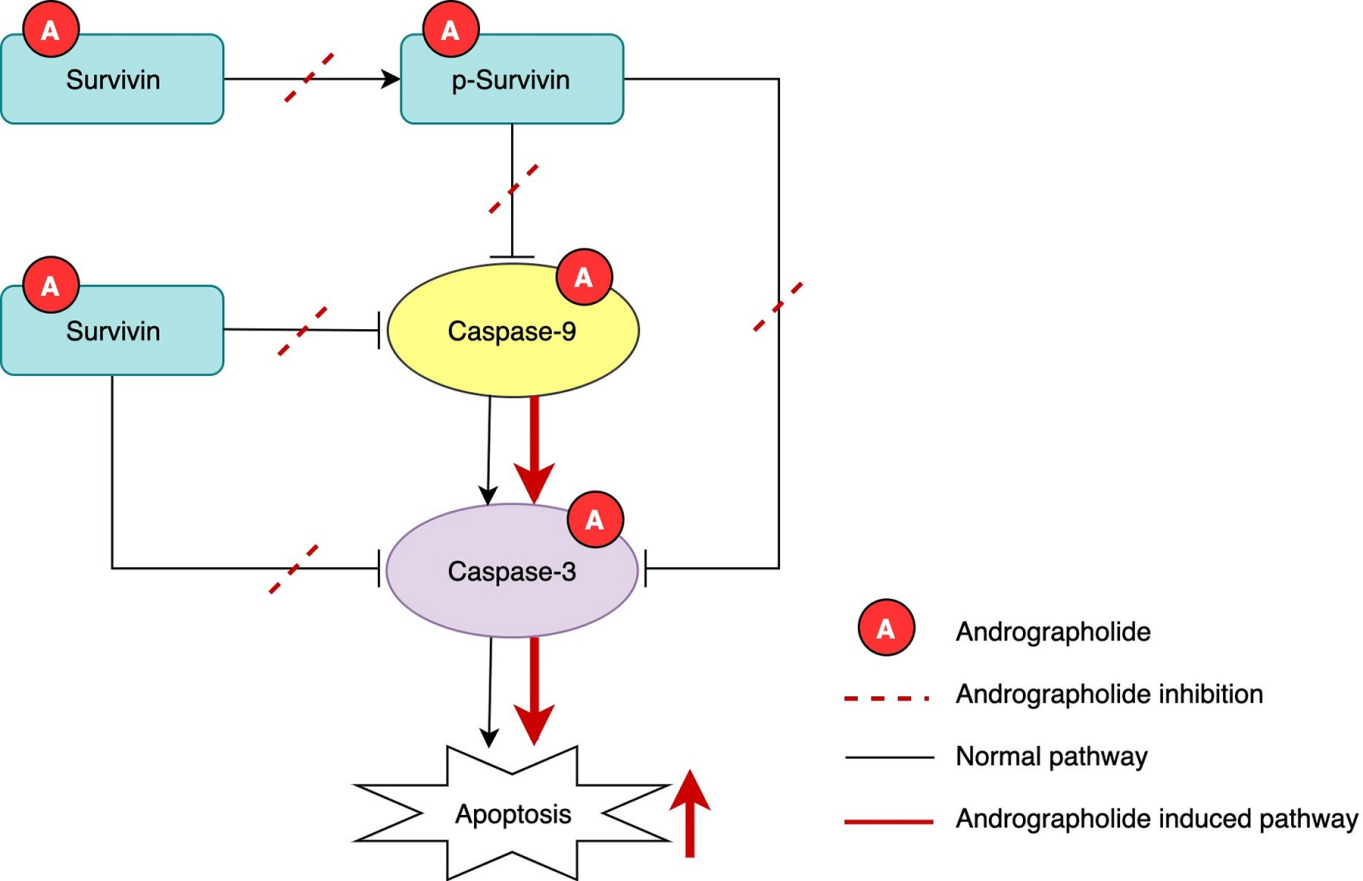
Proposed mechanism of andrographolide on the intrinsic apoptosis pathway. Andrographolide interacts with survivin, phosphorylated survivin, caspase-9, and caspase-3. Andrographolide treatment could inhibit the phosphorylation of survivin and the binding of survivin and p-survivin to caspase-9 and caspase-3, as shown by the dashed red line. As a consequence, intrinsic apoptosis could be induced through activation of caspase-9 and caspase-3, as shown by the continuous red line in the pathway.
