# Supplementary material for: In silico and in vitro studies on the anti-cancer activity of andrographolide targeting survivin in human breast cancer stem cells
Source: PLoS One. 2020 Nov 19;15(11):e0240020. doi: 10.1371/journal.pone.0240020 (PMC7676700; doi:10.1371/journal.pone.0240020)

**S1 Fig. Physicochemical data of seventh active compounds.**

Physicochemical data obtained using SWISSADME server.


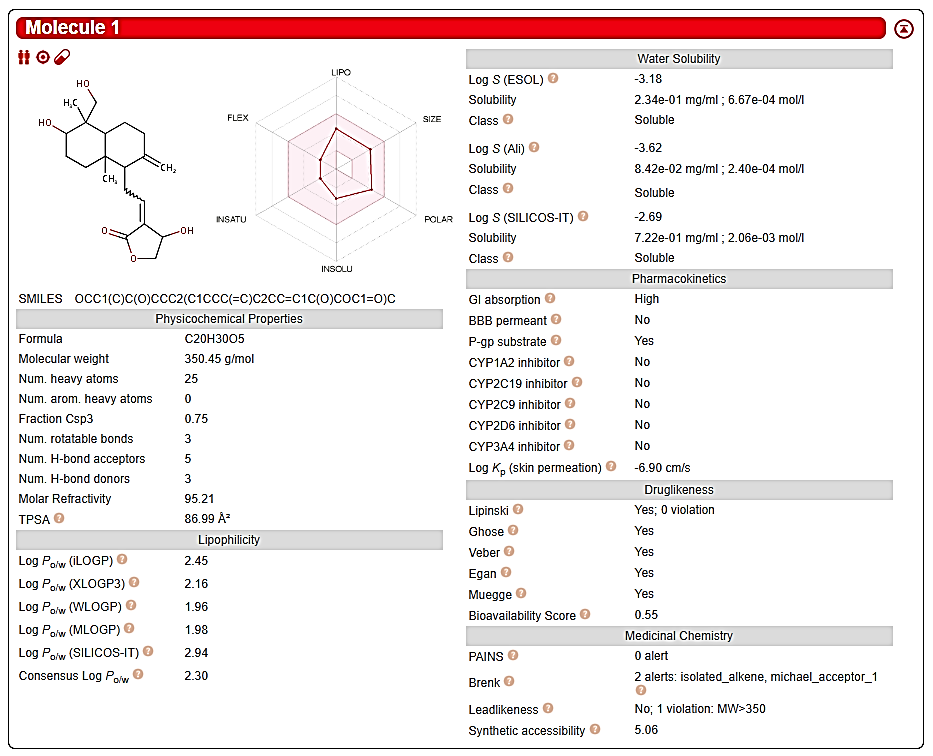


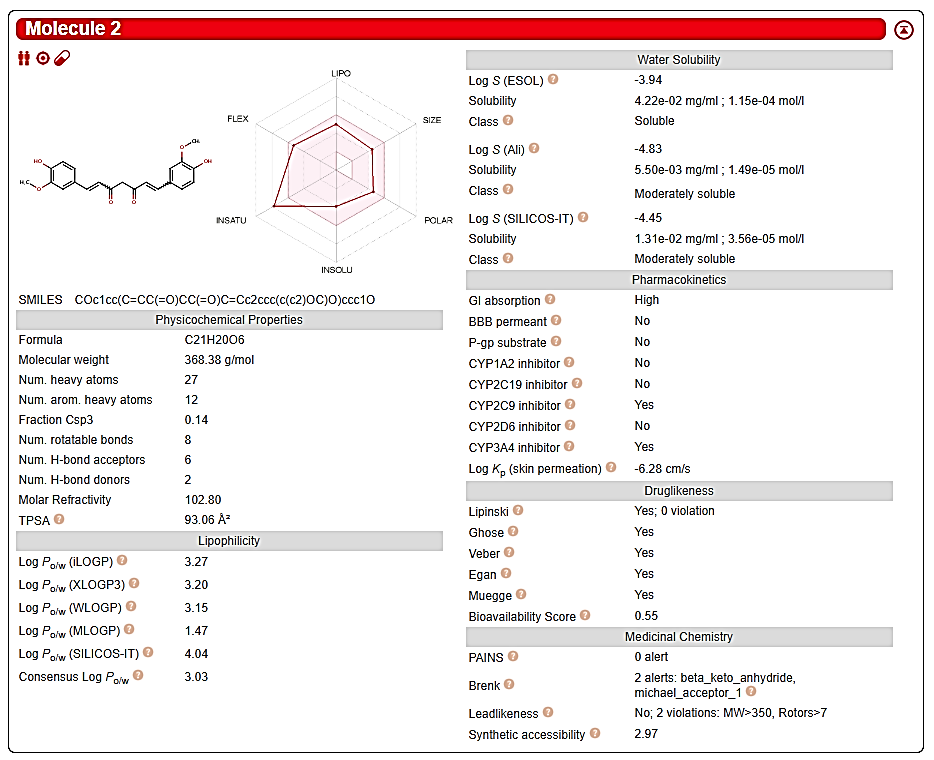


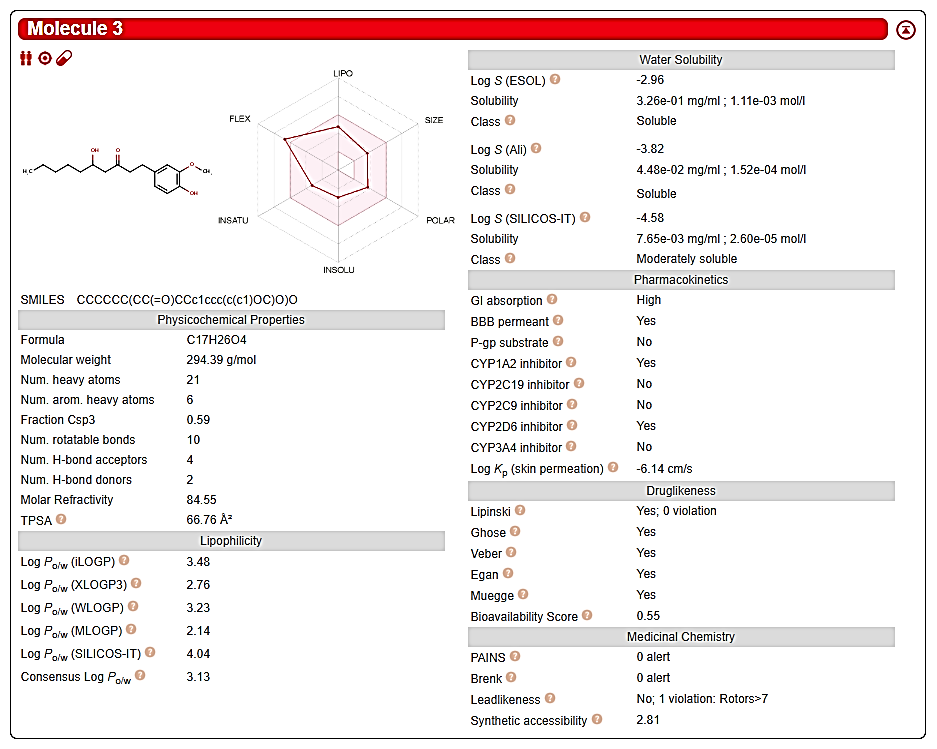


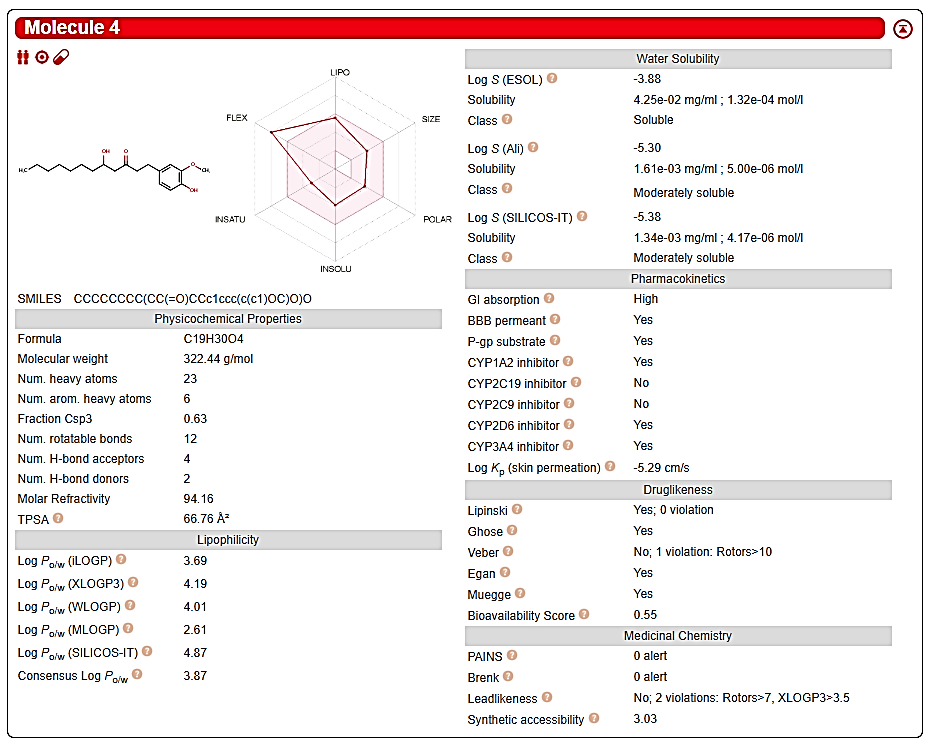


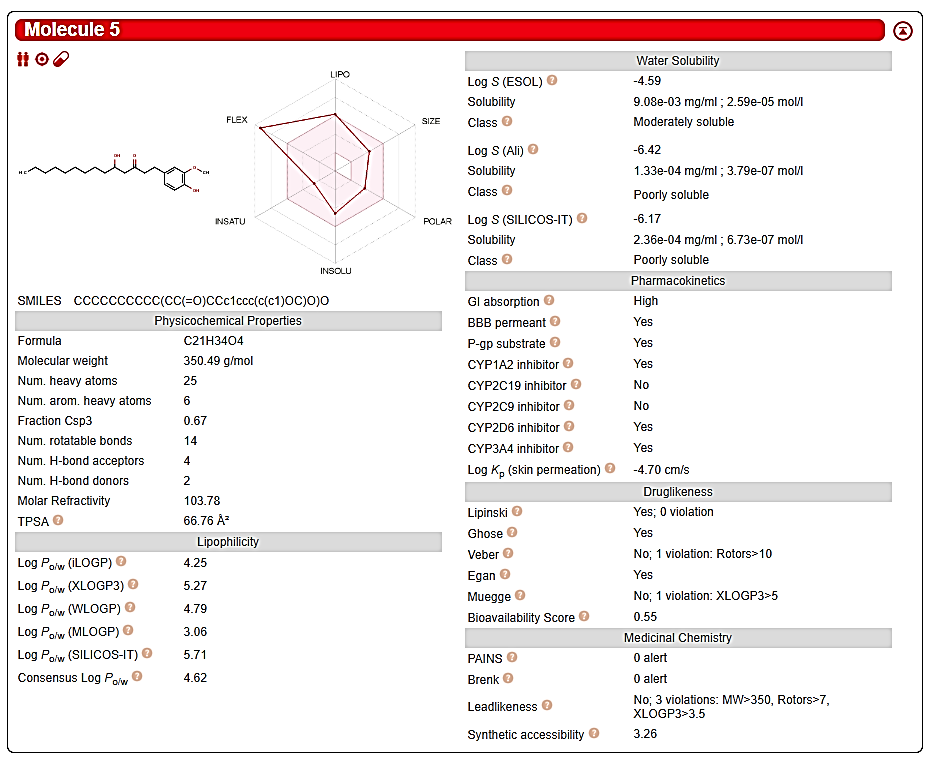


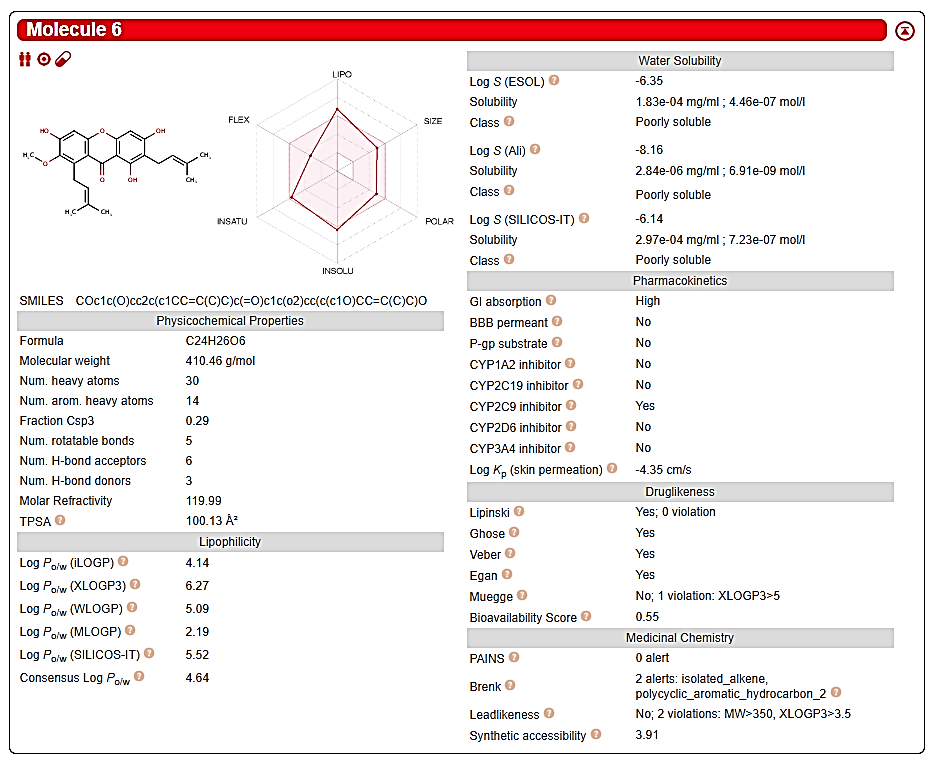


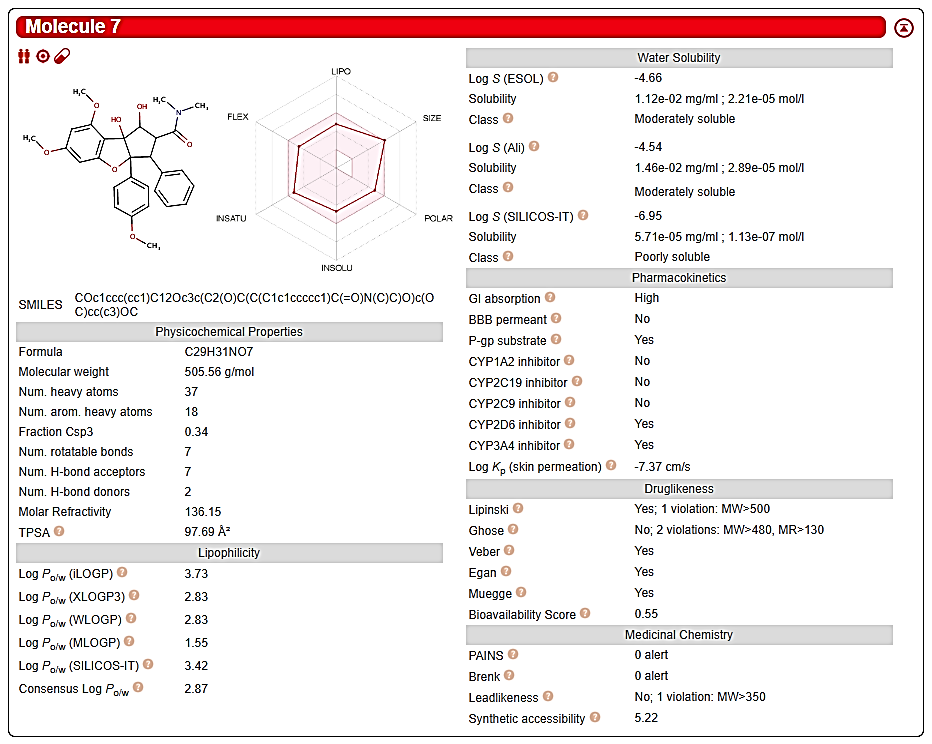

Supplement: S1 Fig — Physicochemical data obtained using SWISSADME server. (DOCX) [file pone.0240020.s001.docx]
