# Supplementary material for: In silico and in vitro studies on the anti-cancer activity of andrographolide targeting survivin in human breast cancer stem cells
Source: PLoS One. 2020 Nov 19;15(11):e0240020. doi: 10.1371/journal.pone.0240020 (PMC7676700; doi:10.1371/journal.pone.0240020)
Supplement: S2 Fig — A total of 100.000 cells were treated with 0.075, 0.15, 0.3, or 0.6 mM andrographolide for 24 hours. Data are presented as mean ± standard deviation (SD) and analyzed using Student’s t-test. The significance levels were shown as **p<0.01 compared to CD24-/CD44+ cells. (DOCX) [file pone.0240020.s002.docx]

**S2 Fig.** **Cytotoxic activity of andrographolide in human CD24-/CD44- breast cancer cells (non-BCSCs), and human MCF-7 breast cancer cell line compared to that in human BCSCs.** A total of 100.000 cells were treated with 0.075, 0.15, 0.3, or 0.6 mM andrographolide for 24 hours. Data are presented as mean ± standard deviation (SD) and analyzed using Student's t-test. The significance levels were shown as **p<0.01 compared to CD24-/CD44+ cells.

**
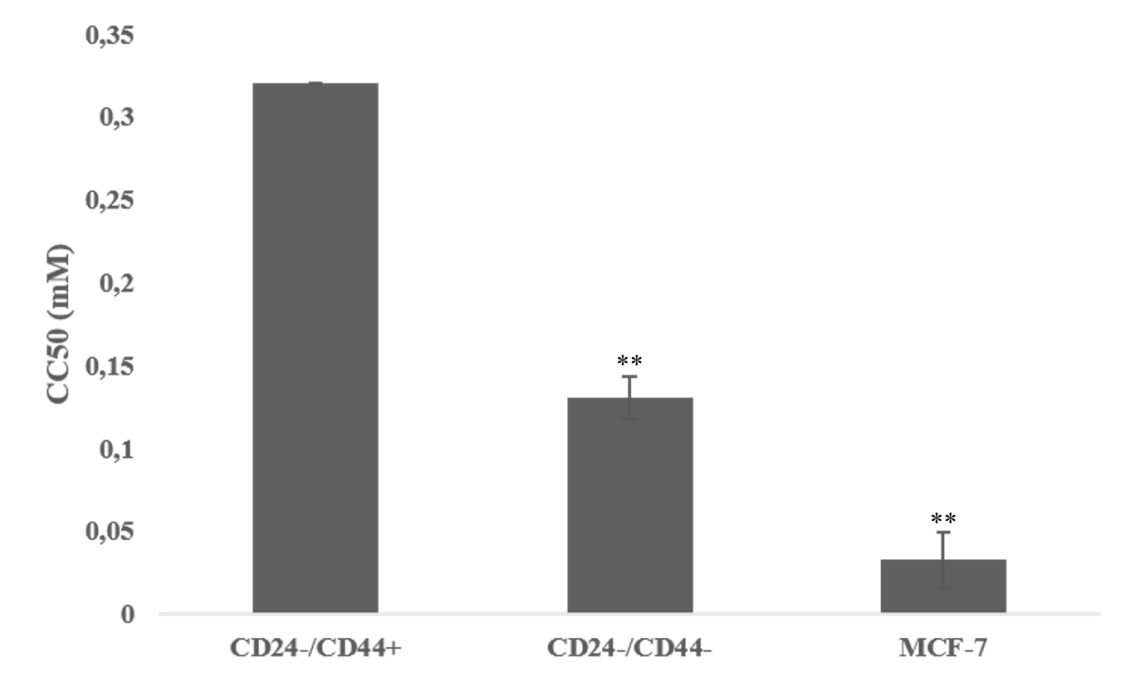
**
