# Supplementary material for: In silico and in vitro studies on the anti-cancer activity of andrographolide targeting survivin in human breast cancer stem cells
Source: PLoS One. 2020 Nov 19;15(11):e0240020. doi: 10.1371/journal.pone.0240020 (PMC7676700; doi:10.1371/journal.pone.0240020)
Supplement: S3 Fig — A total of 100.000 cells were treated with 100nM YM155, a survivin inhibitor, at Day 0, 2, and 4. Cells were then harvested and counted every day. Data are presented as mean ± standard deviation (SD) and analyzed using Student’s t-test. The statistical significance levels were shown as **p<0.01 and *p<0.05 compared to its respective control without YM155. (DOCX) [file pone.0240020.s003.docx]

**S3 Fig.** **Proliferation rate of human CD24-/CD44+ BCSCs with 100nM YM155, a survivin inhibitor.** A total of 100.000 cells were treated with 100nM YM155, a survivin inhibitor, at Day 0, 2, and 4. Cells were then harvested and counted every day. Data are presented as mean ± standard deviation (SD) and analyzed using Student's t-test. The statistical significance levels were shown as **p<0.01 and *p<0.05 compared to its respective control without YM155.
