# Supplementary figures and images for: In silico and in vitro studies on the anti-cancer activity of andrographolide targeting survivin in human breast cancer stem cells
Source: PLoS One. 2020 Nov 19;15(11):e0240020. doi: 10.1371/journal.pone.0240020 (PMC7676700; doi:10.1371/journal.pone.0240020)

**S4 Fig. Melting curve qRT-PCR 18S rRNA, survivin, caspase-9, and caspase-3**

**18S rRNA**

**
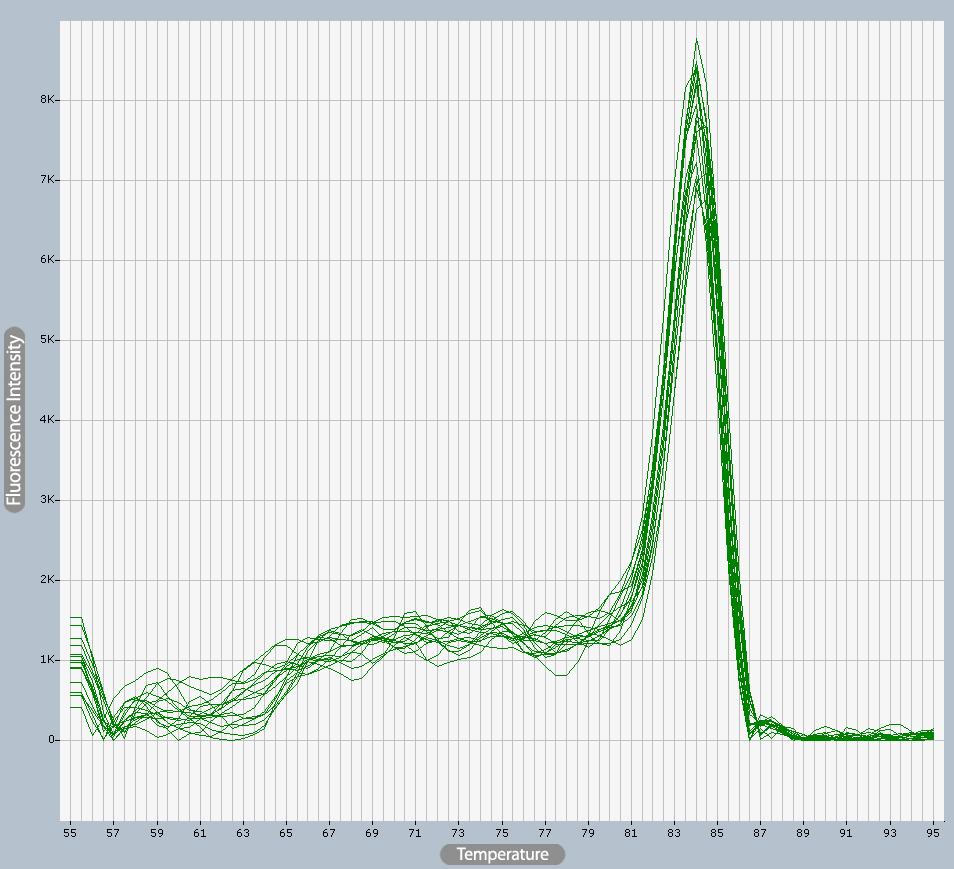
**

**Survivin**

**
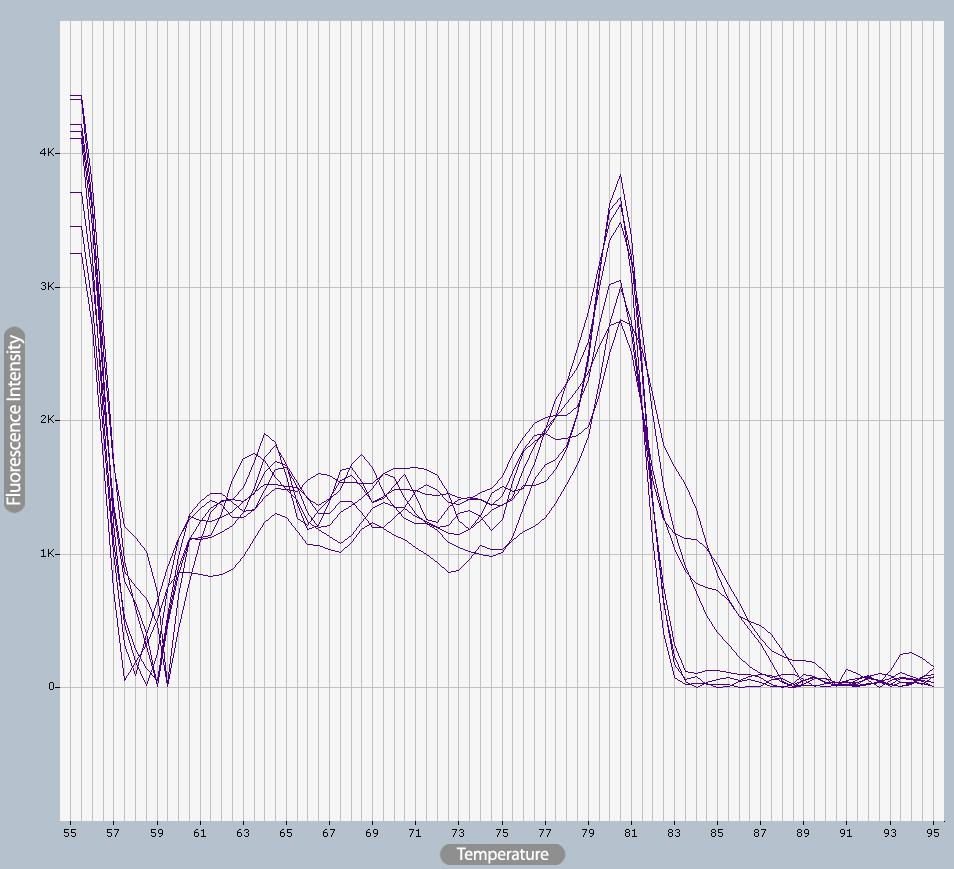
**

**Caspase-9**

**
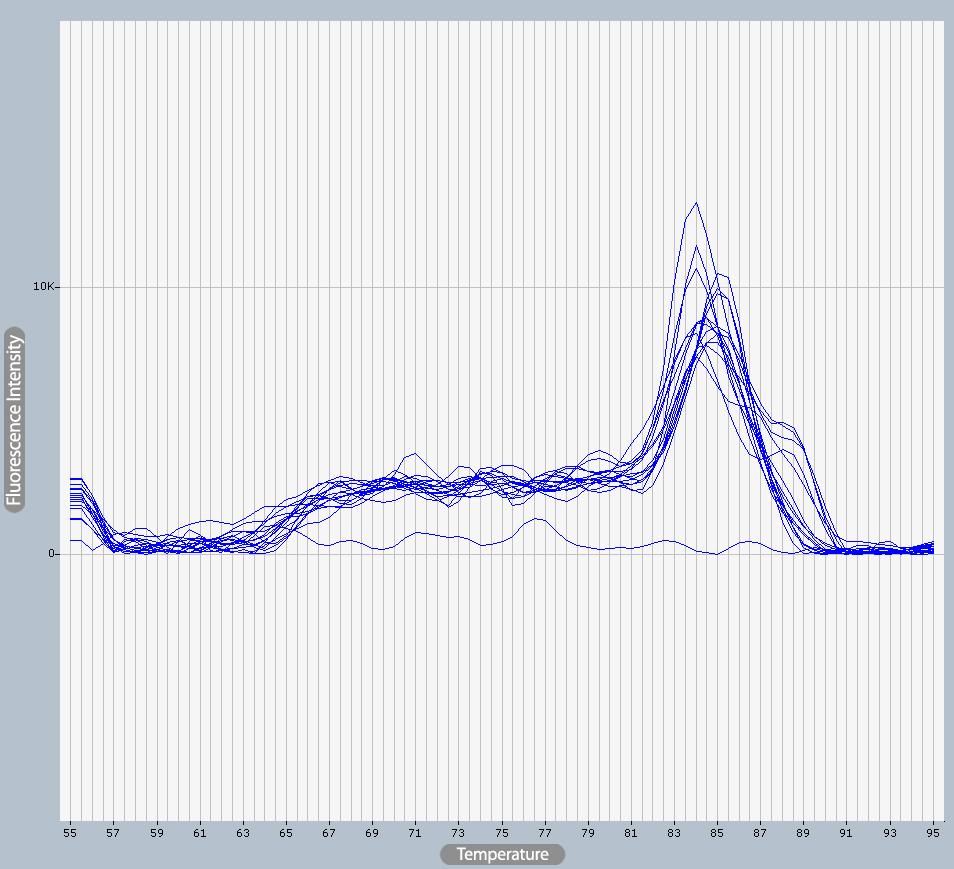
**

**Caspase-3**

**
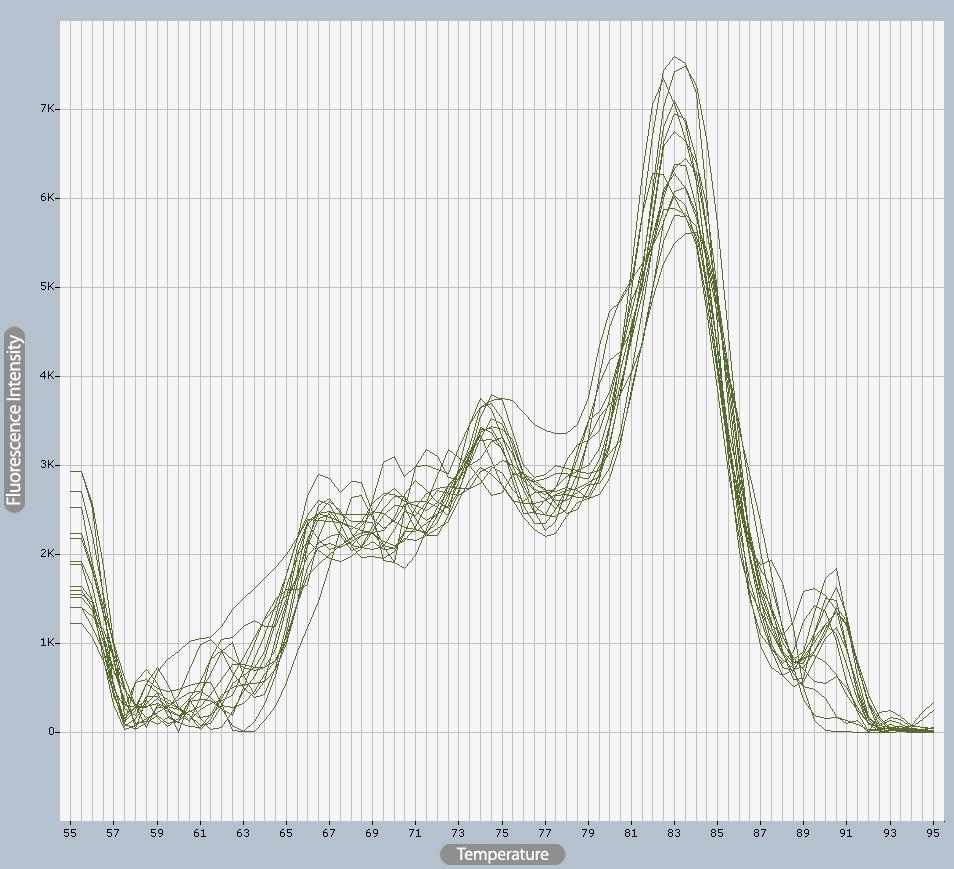
**

Supplement: S4 Fig — (DOCX) [file pone.0240020.s004.docx]
