# Supplementary material for: In silico and in vitro studies on the anti-cancer activity of andrographolide targeting survivin in human breast cancer stem cells
Source: PLoS One. 2020 Nov 19;15(11):e0240020. doi: 10.1371/journal.pone.0240020 (PMC7676700; doi:10.1371/journal.pone.0240020)

**S5 Fig. Statistical analysis of qRT-PCR and ELISA data using SPSS software version 26.**

1. **Survivin mRNA expression**


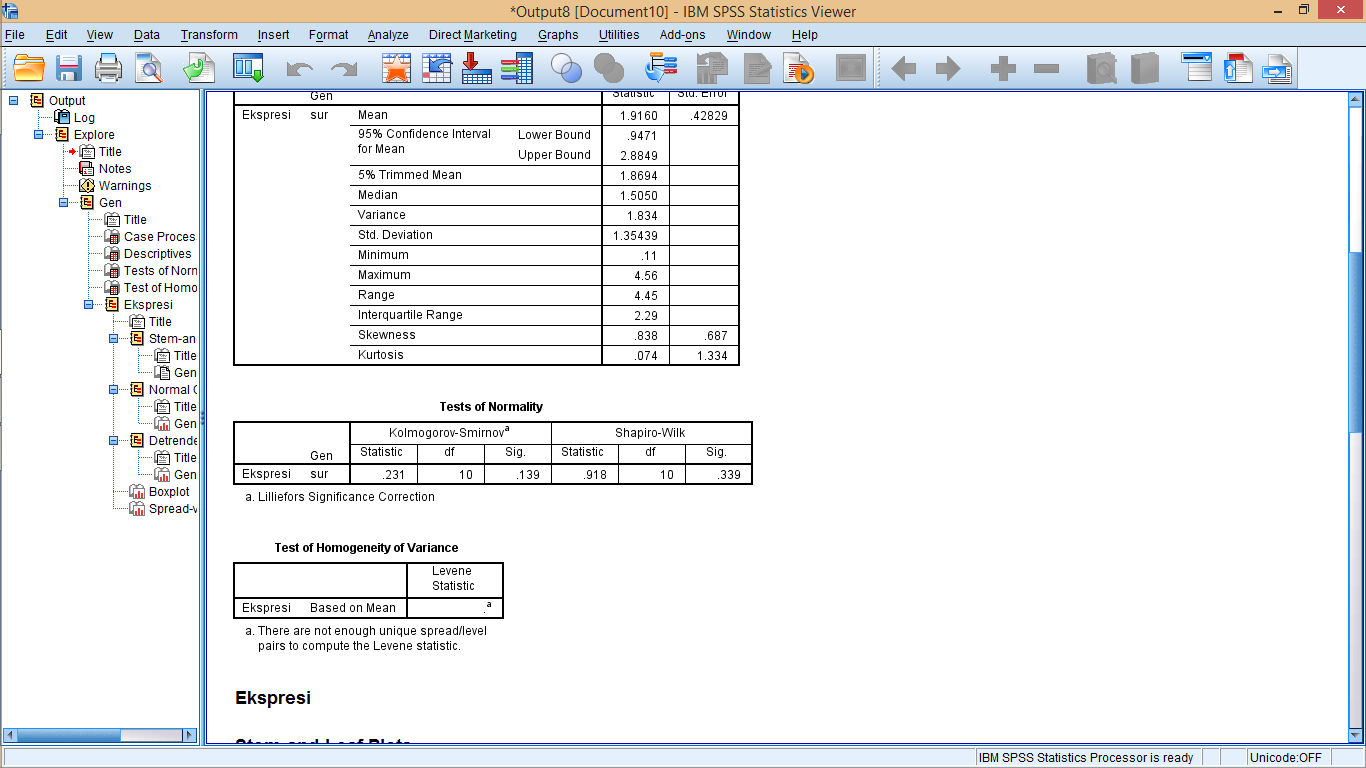


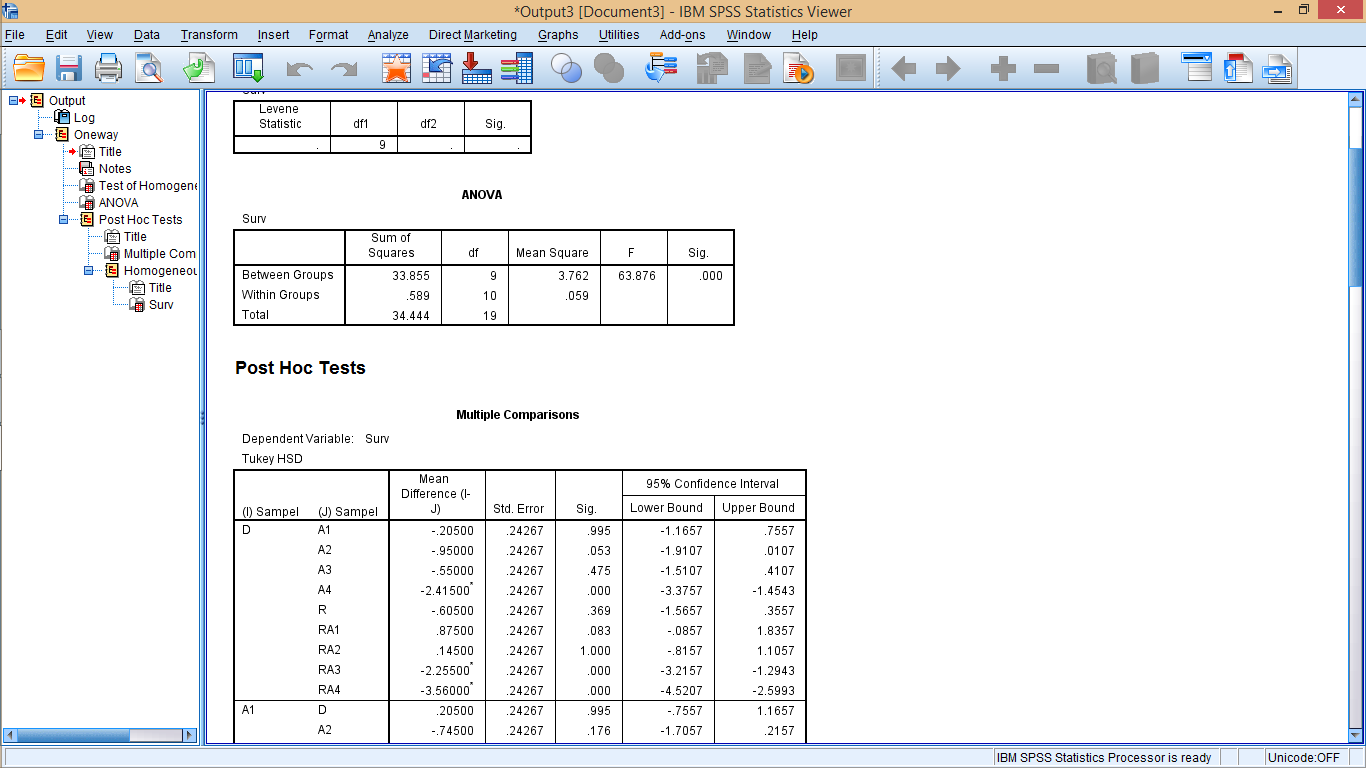


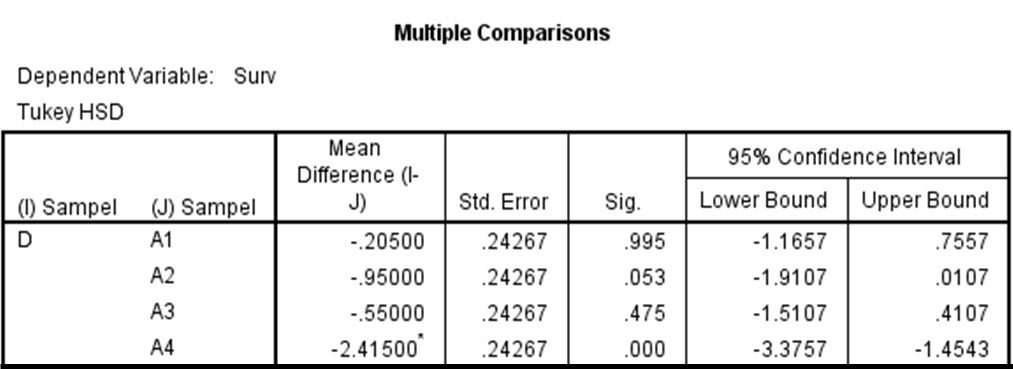


1. **Caspase 9 mRNA expression**


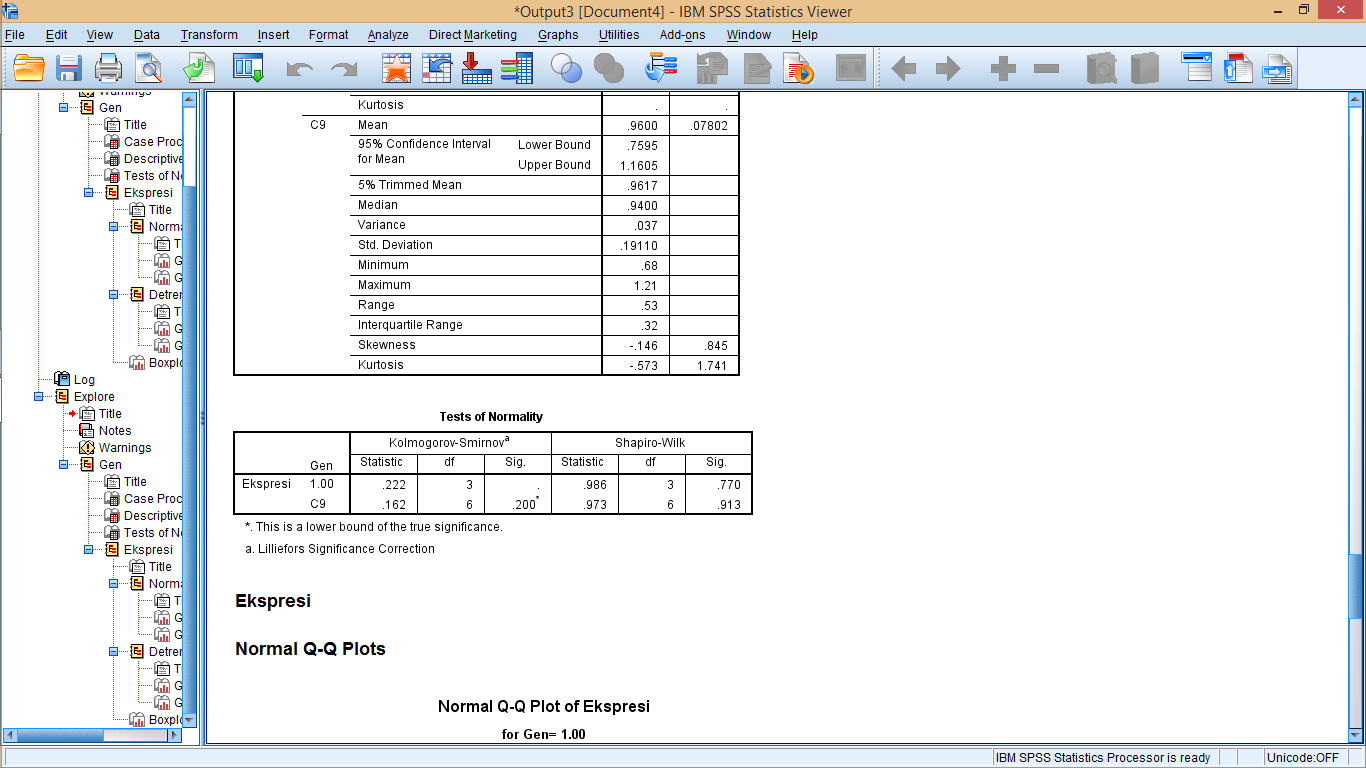


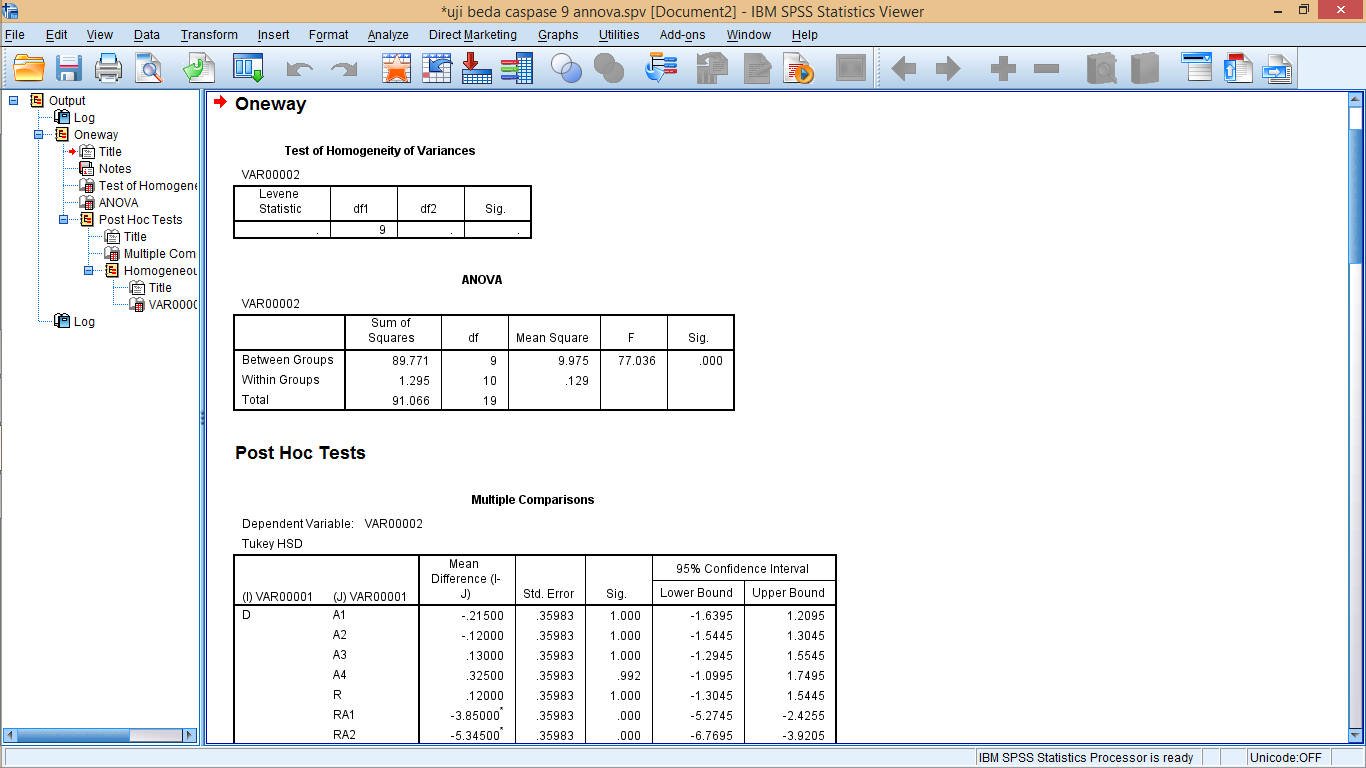


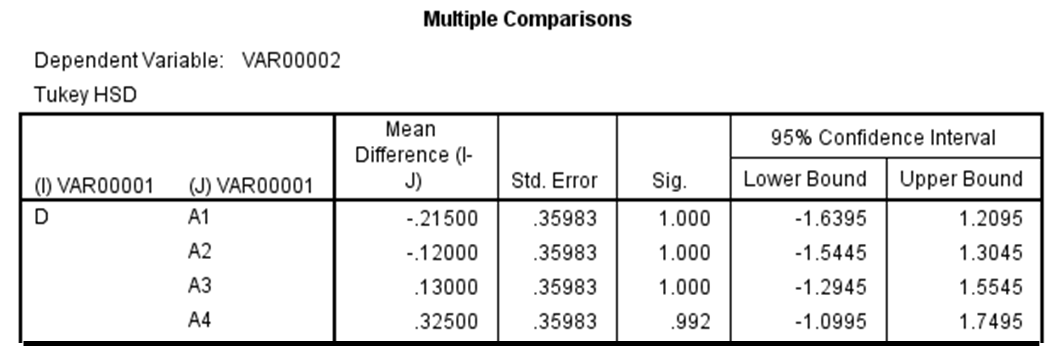


1. **Caspase 3 mRNA expression**


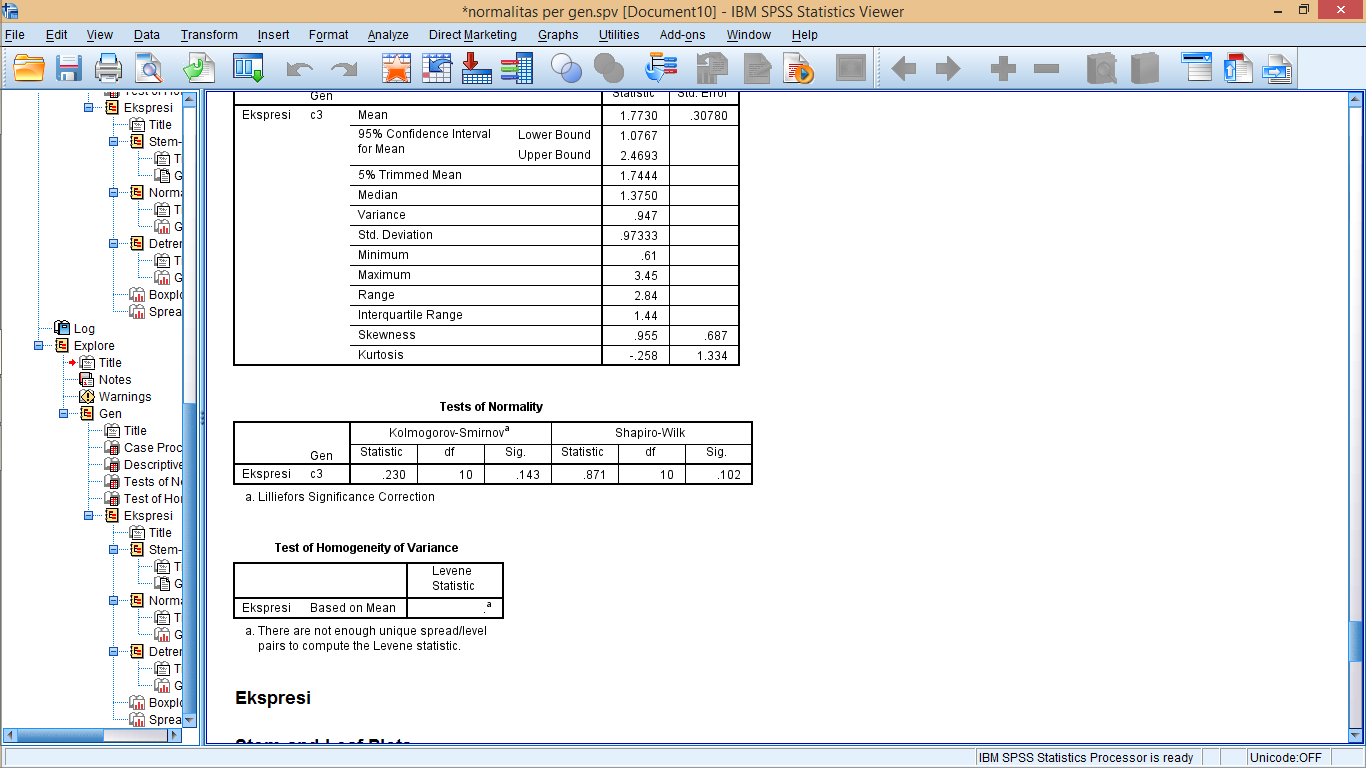


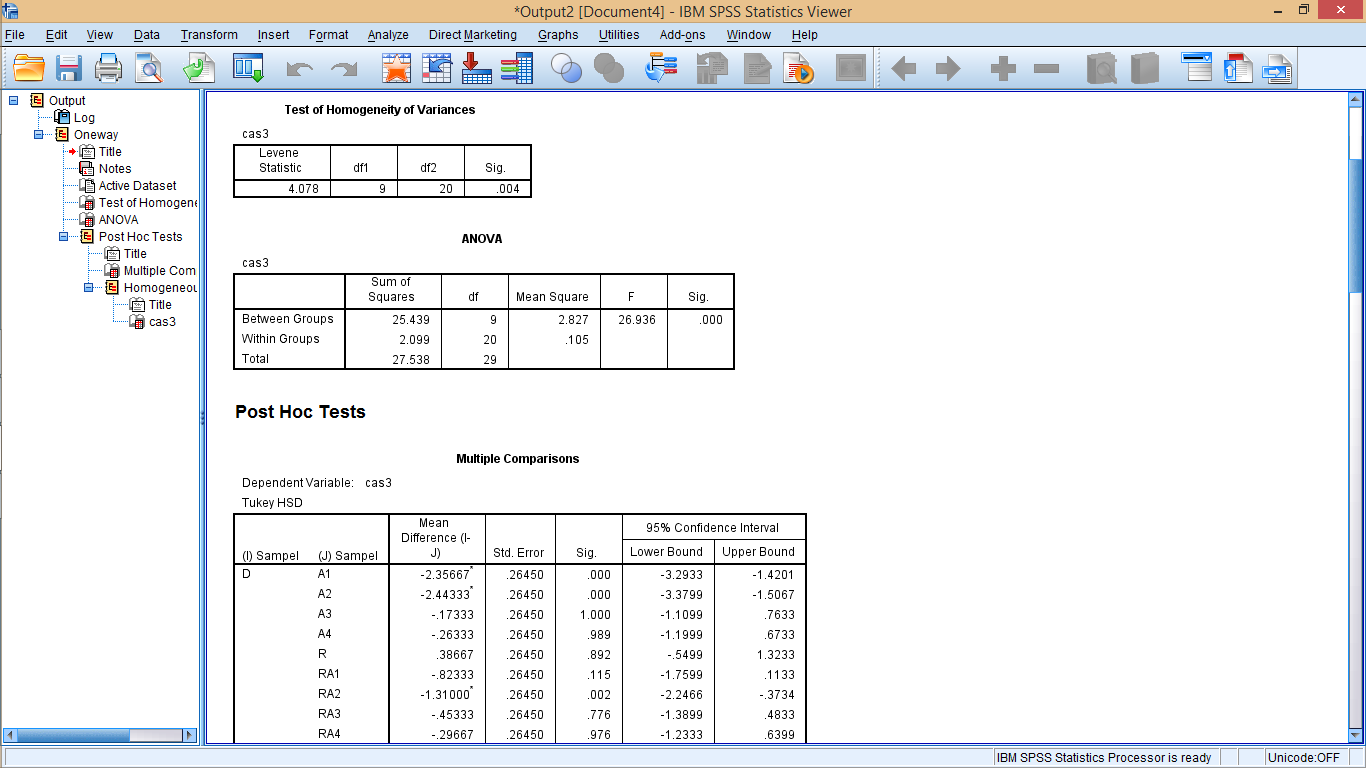


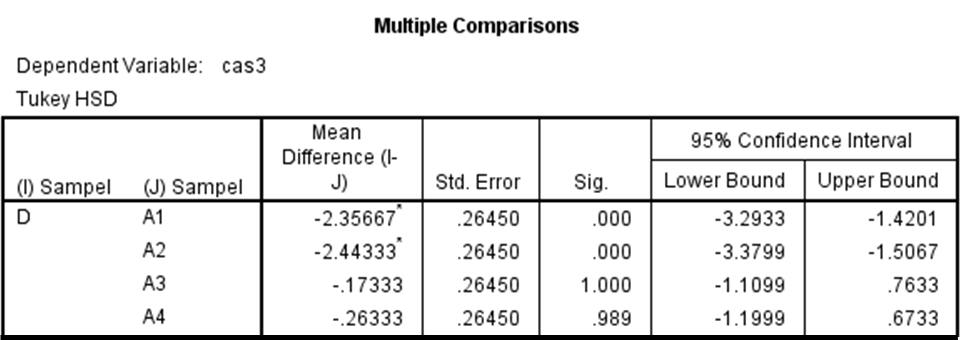


1. **Total survivin protein level**


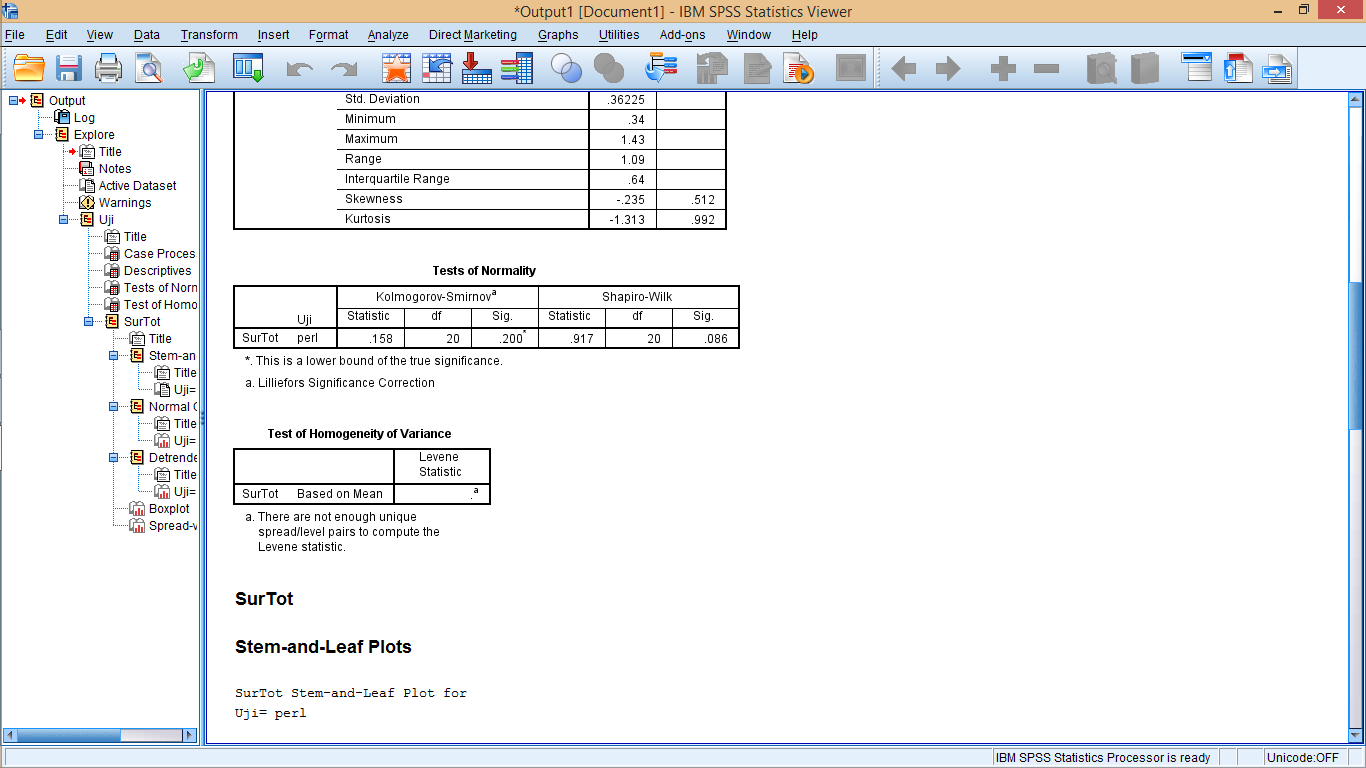


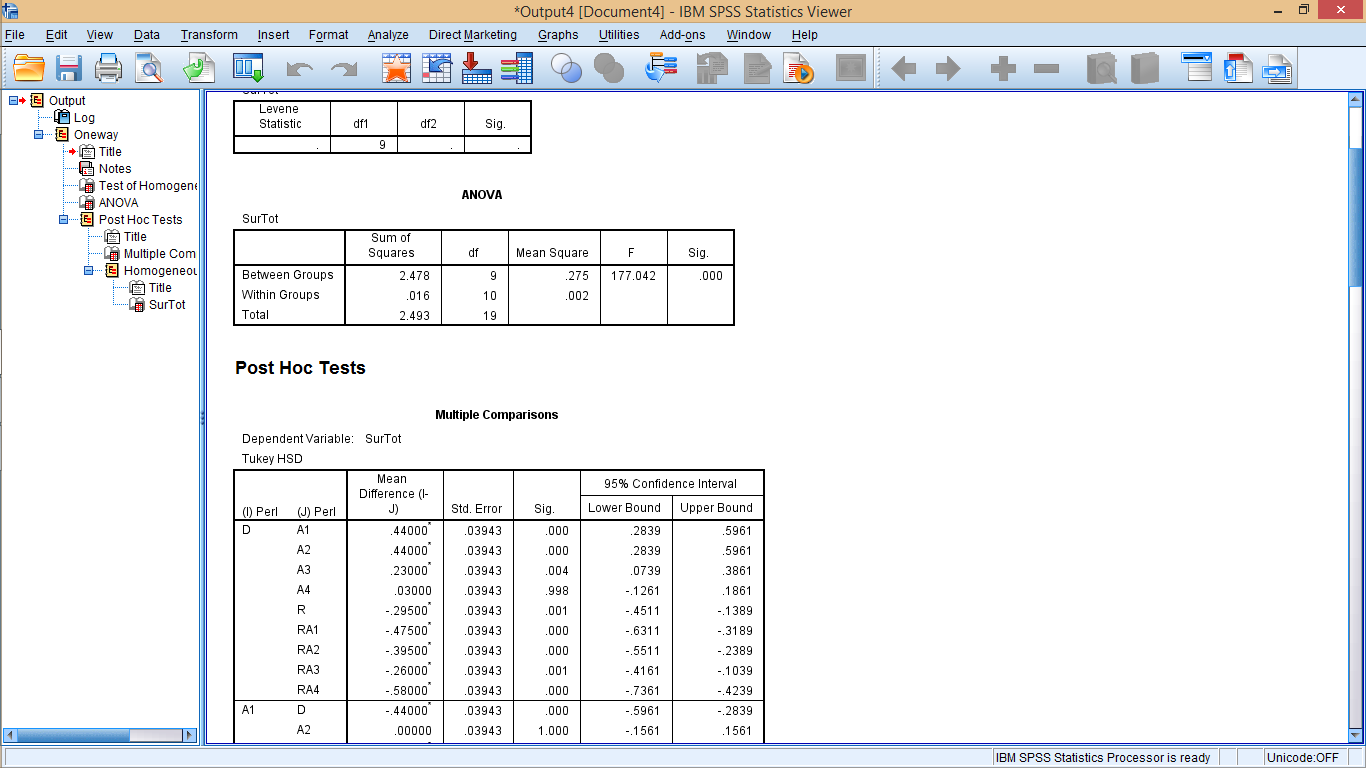


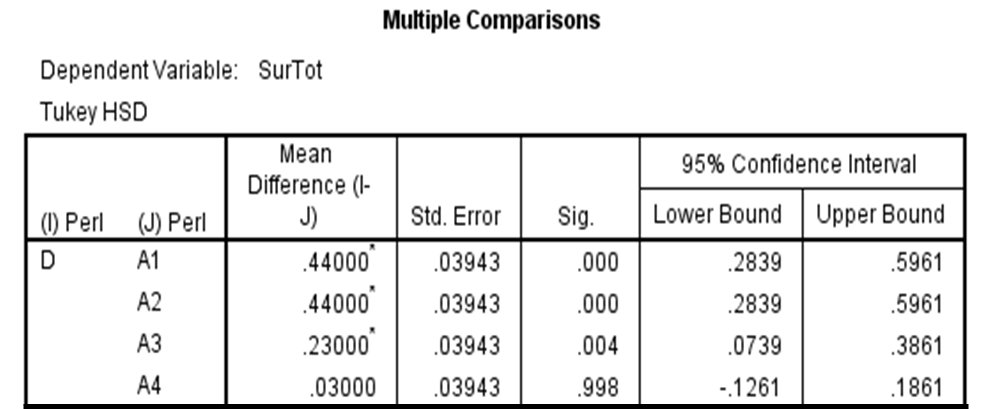


1. **Phosphorylated survivin protein level**


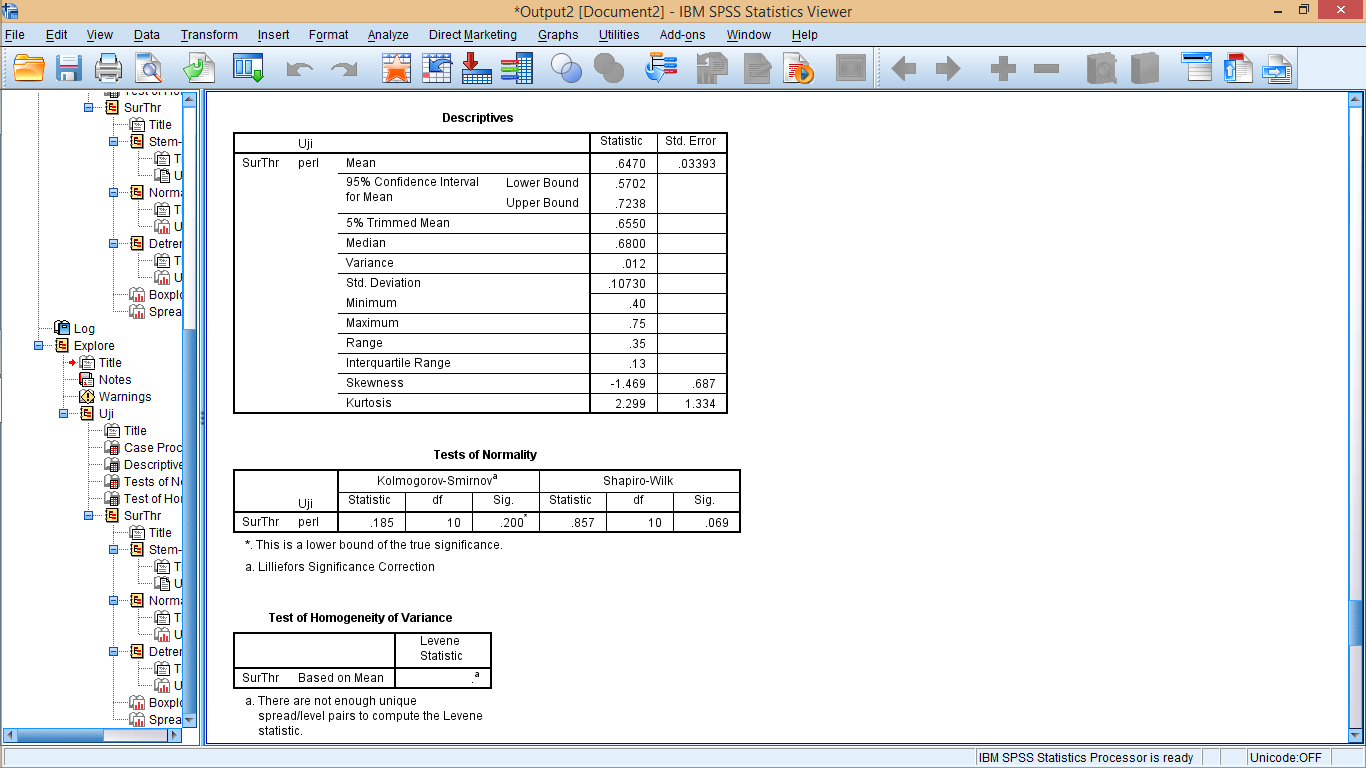


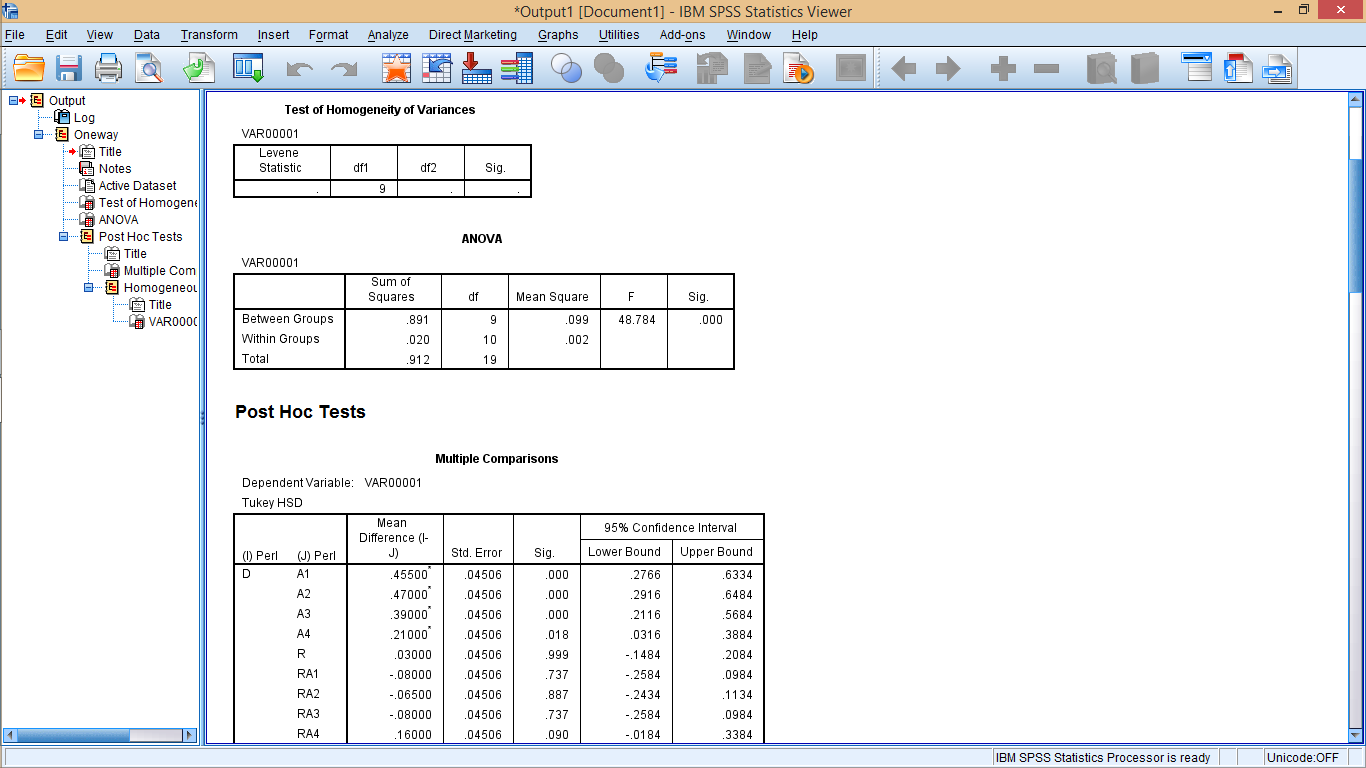


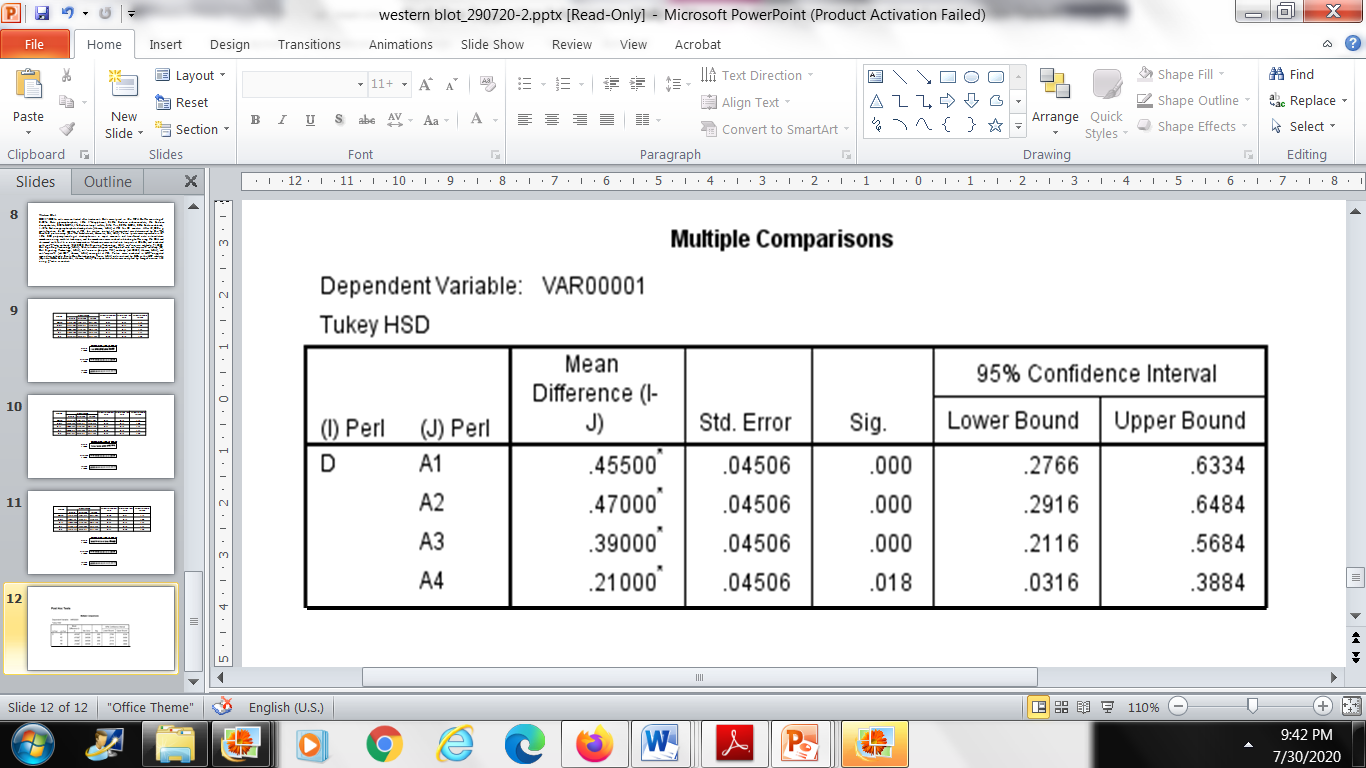

Supplement: S5 Fig — (DOCX) [file pone.0240020.s005.docx]
