# Supplementary material for: In silico and in vitro studies on the anti-cancer activity of andrographolide targeting survivin in human breast cancer stem cells
Source: PLoS One. 2020 Nov 19;15(11):e0240020. doi: 10.1371/journal.pone.0240020 (PMC7676700; doi:10.1371/journal.pone.0240020)
Supplement: S6 Fig — Membrane was first used for immunoblotting with anti caspase-3 antibody. Then, the membrane was horizontally cut into 2 parts at 30 kDa level and stripped for immunoblotting with anti-beta actin, anti-survivin, anti-caspase-9, and anti-phospho survivin. (DOCX) [file pone.0240020.s006.docx]

**S6 Fig. Immunoblot of survivin, caspase-9, caspase-3, and beta actin.** Membrane was first used for immunoblotting with anti caspase-3 antibody. Then, the membrane was horizontally cut into 2 parts at 30 kDa level and stripped for immunoblotting with anti-beta actin, anti-survivin, anti-caspase-9, and anti-phospho survivin.

**
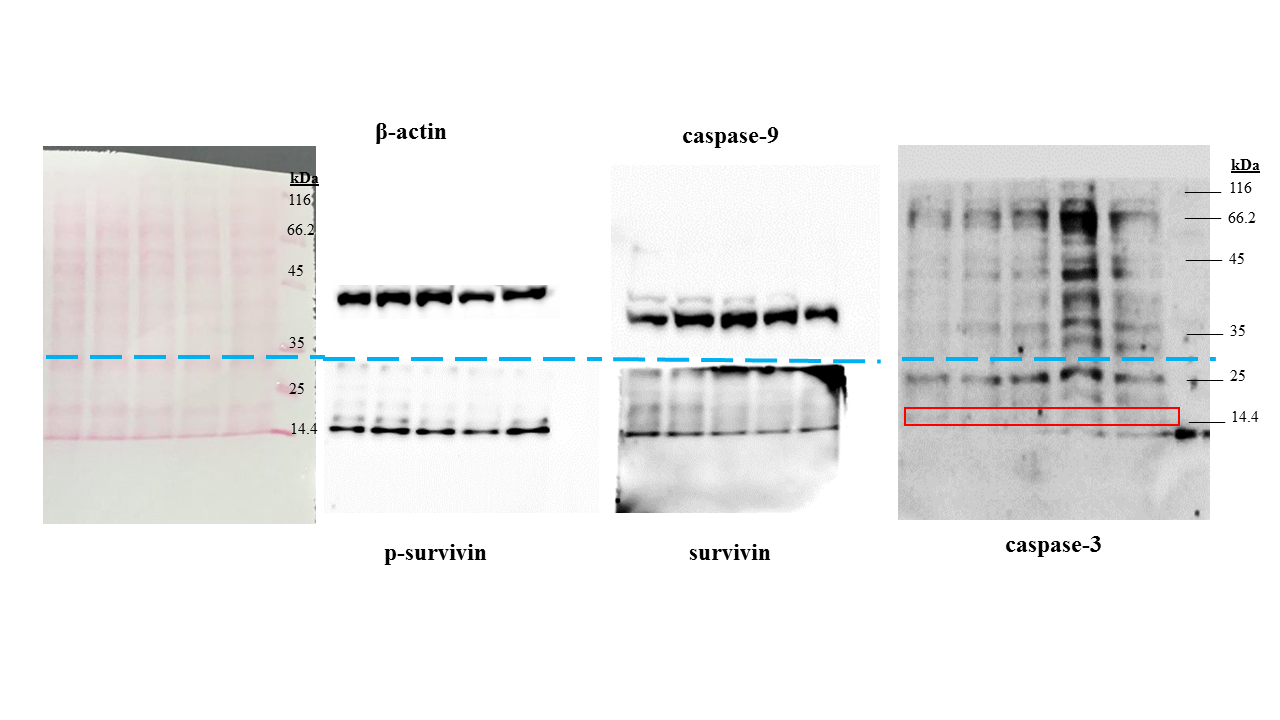
**
