# Supplementary material for: In silico and in vitro studies on the anti-cancer activity of andrographolide targeting survivin in human breast cancer stem cells
Source: PLoS One. 2020 Nov 19;15(11):e0240020. doi: 10.1371/journal.pone.0240020 (PMC7676700; doi:10.1371/journal.pone.0240020)

**S7 Fig. Data of apoptosis analysis of andrographolide-treated BCSCs using flow cytometry. (**Output Data from flow cytometry Instrument)

**Control**


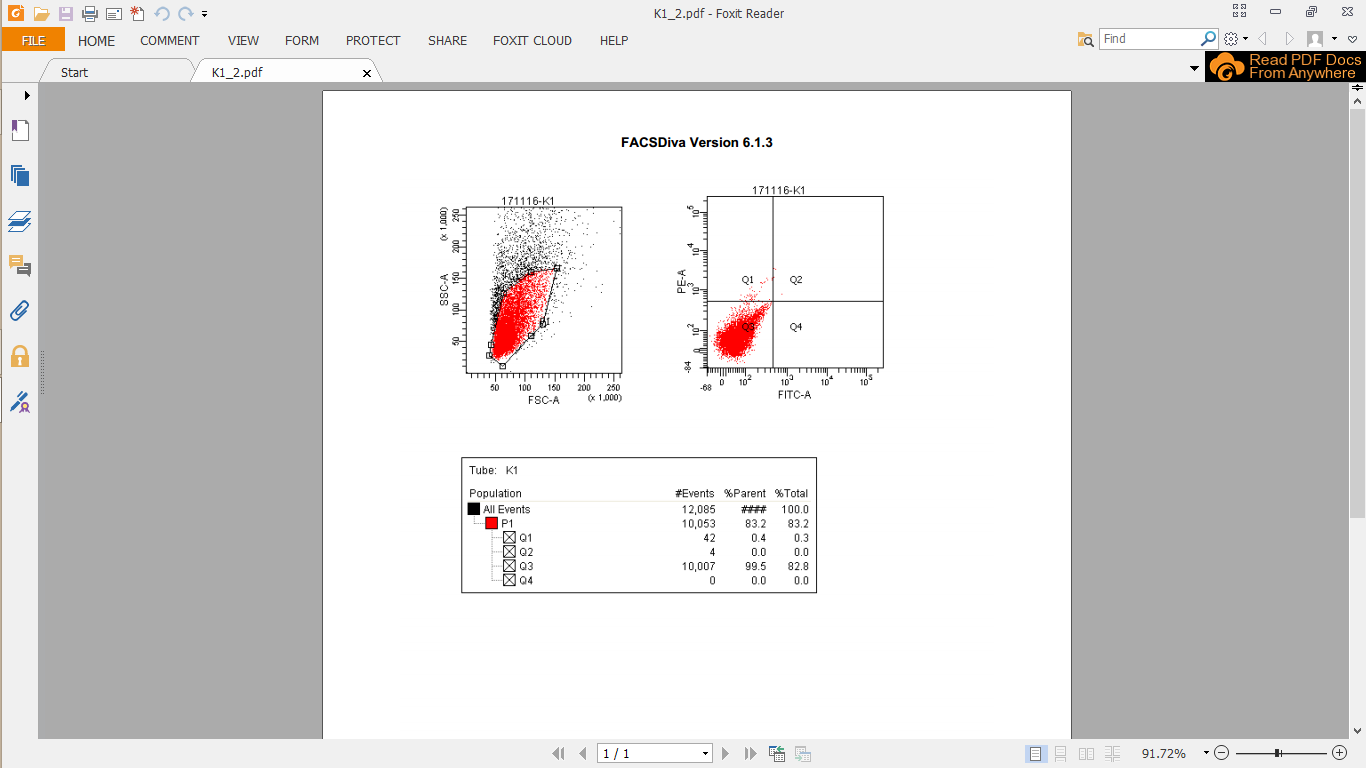


**Control (DMSO 0.01%)**


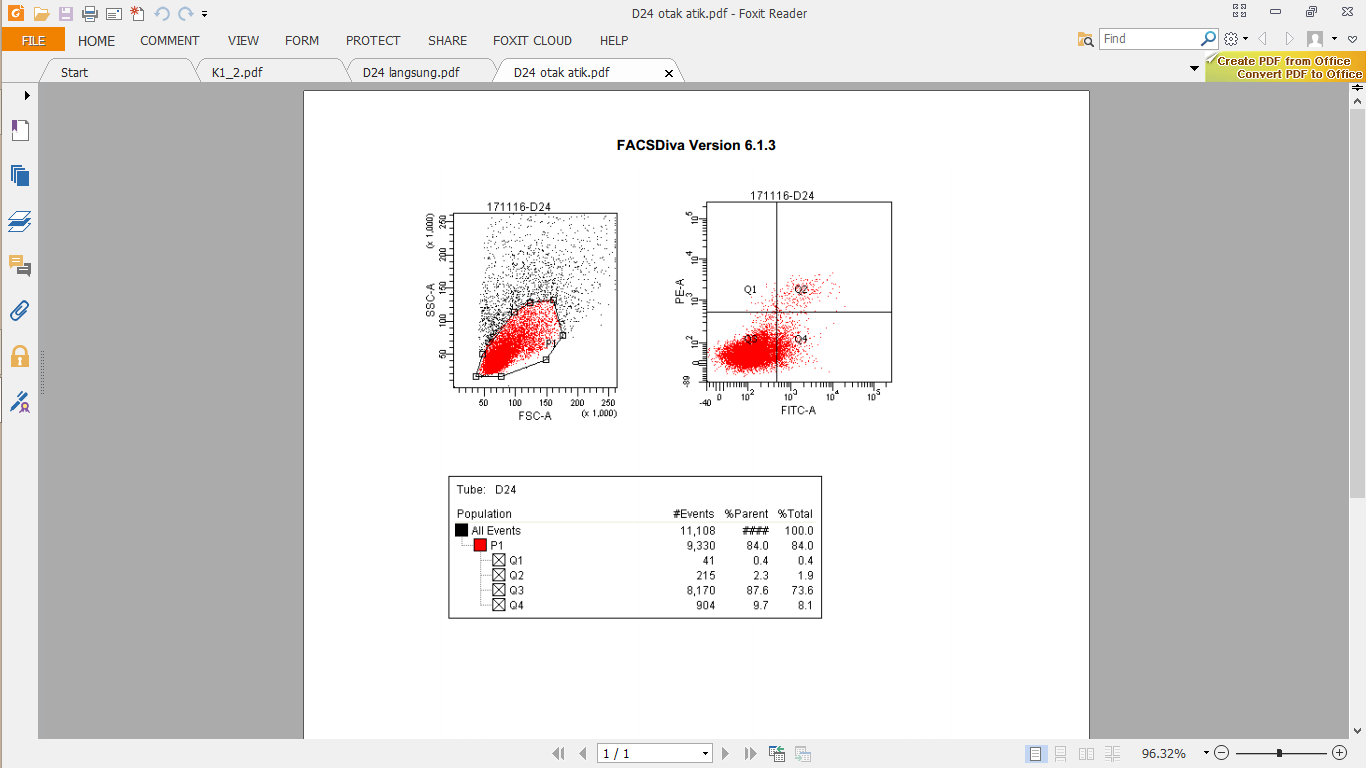


**ANDRO 0.075 mM**


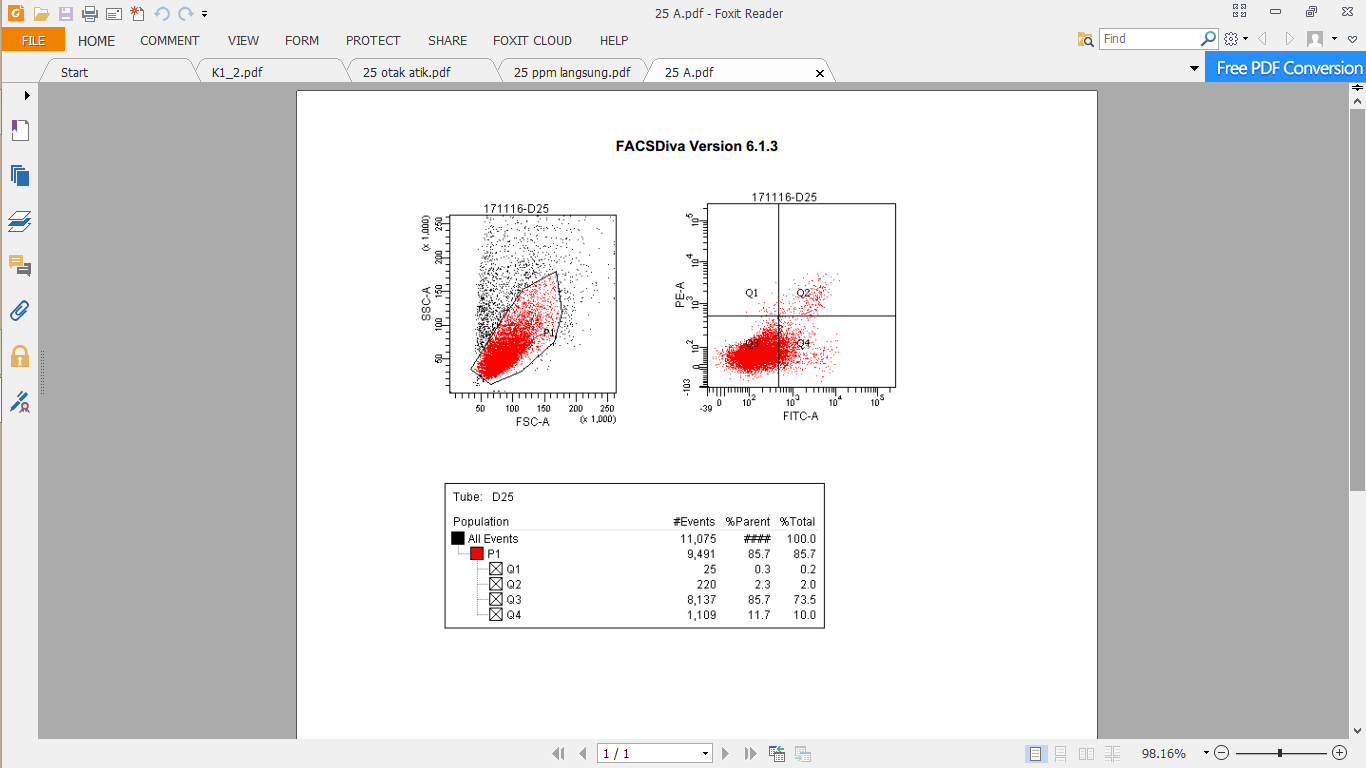


**ANDRO 0.15 mM**


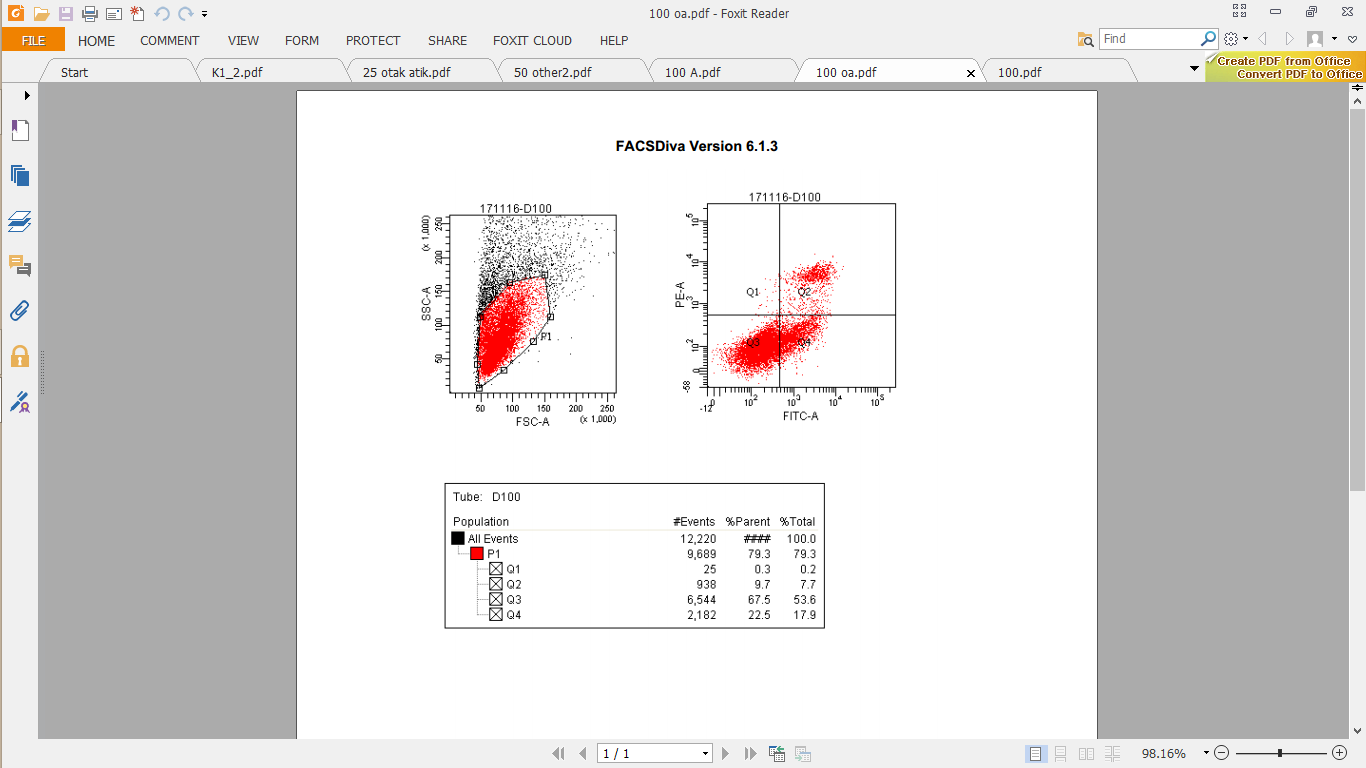


**ANDRO 0.3 mM**


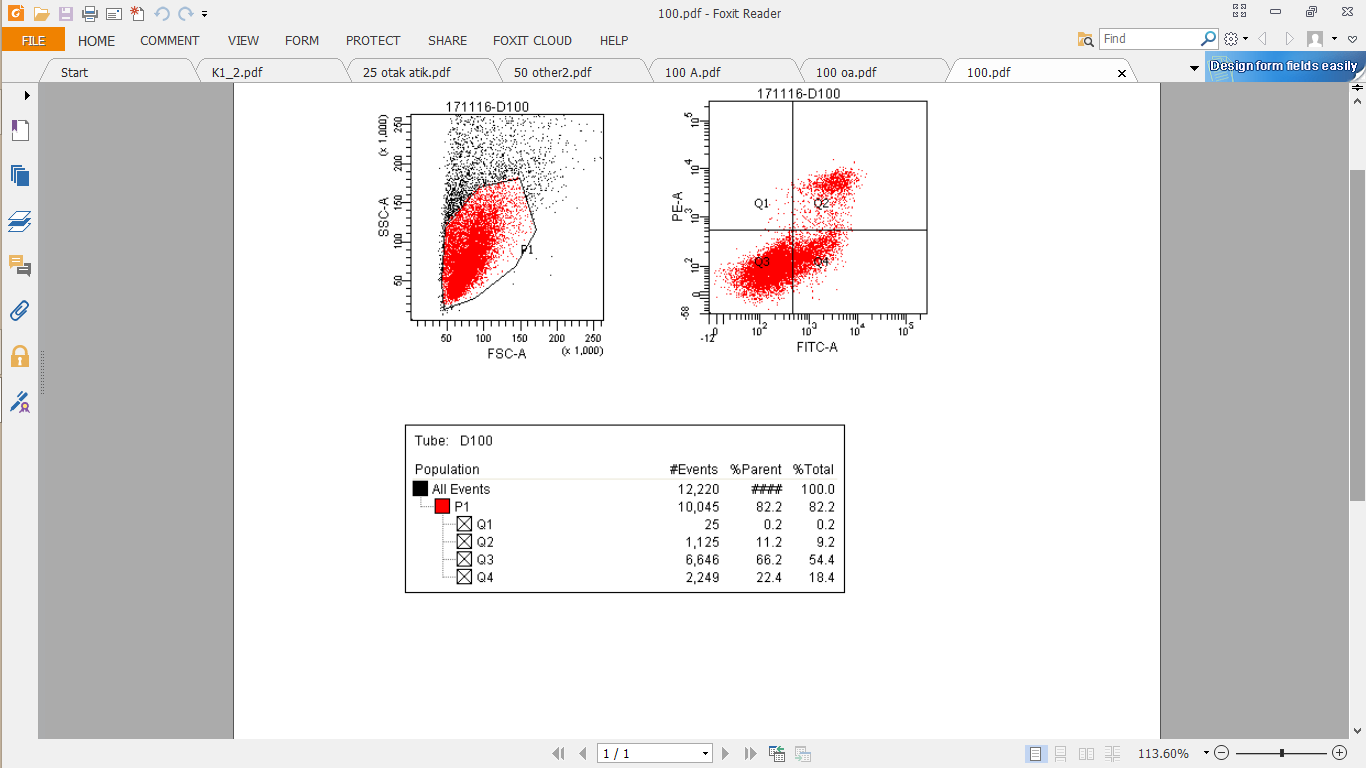


**ANDRO 0.6 mM**


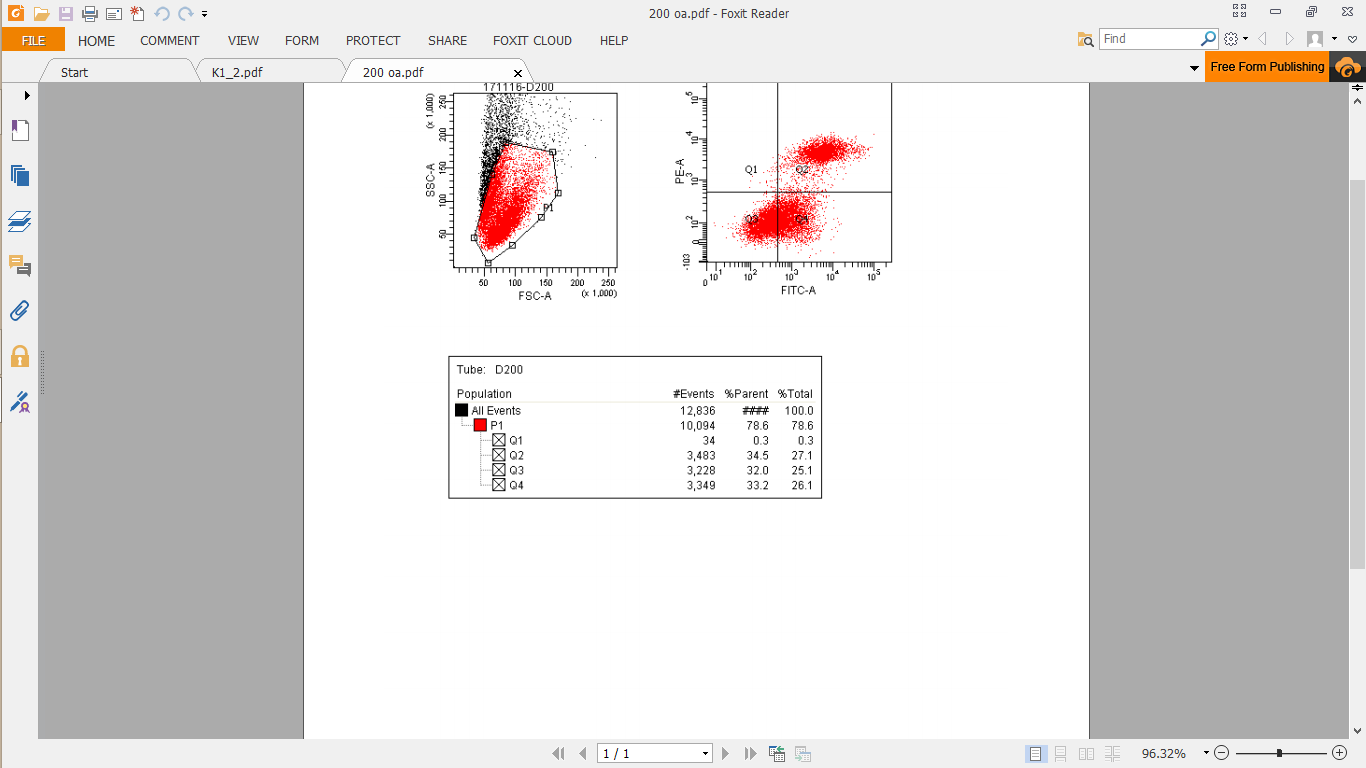

Supplement: S7 Fig — (Output Data from flow cytometry Instrument). (DOCX) [file pone.0240020.s007.docx]
