# Supplementary material for: In silico and in vitro studies on the anti-cancer activity of andrographolide targeting survivin in human breast cancer stem cells
Source: PLoS One. 2020 Nov 19;15(11):e0240020. doi: 10.1371/journal.pone.0240020 (PMC7676700; doi:10.1371/journal.pone.0240020)

**S9A Fig. Protein-protein docking of Survivin and Caspase-3.** Protein-protein docking of survivin (brown) and caspase-3 (yellow) was done by PatchDock Server and the result was refined by FireDock Server.


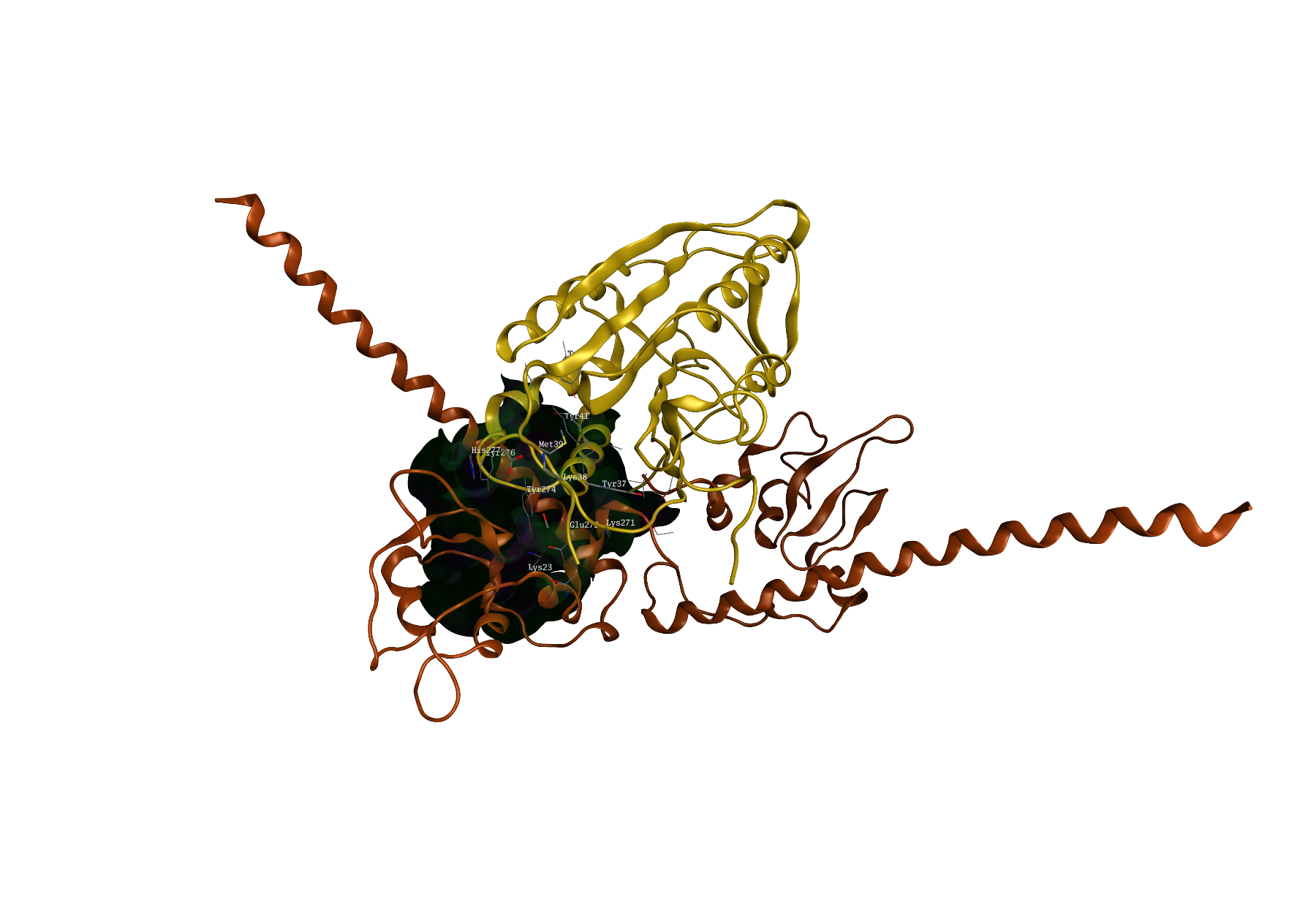


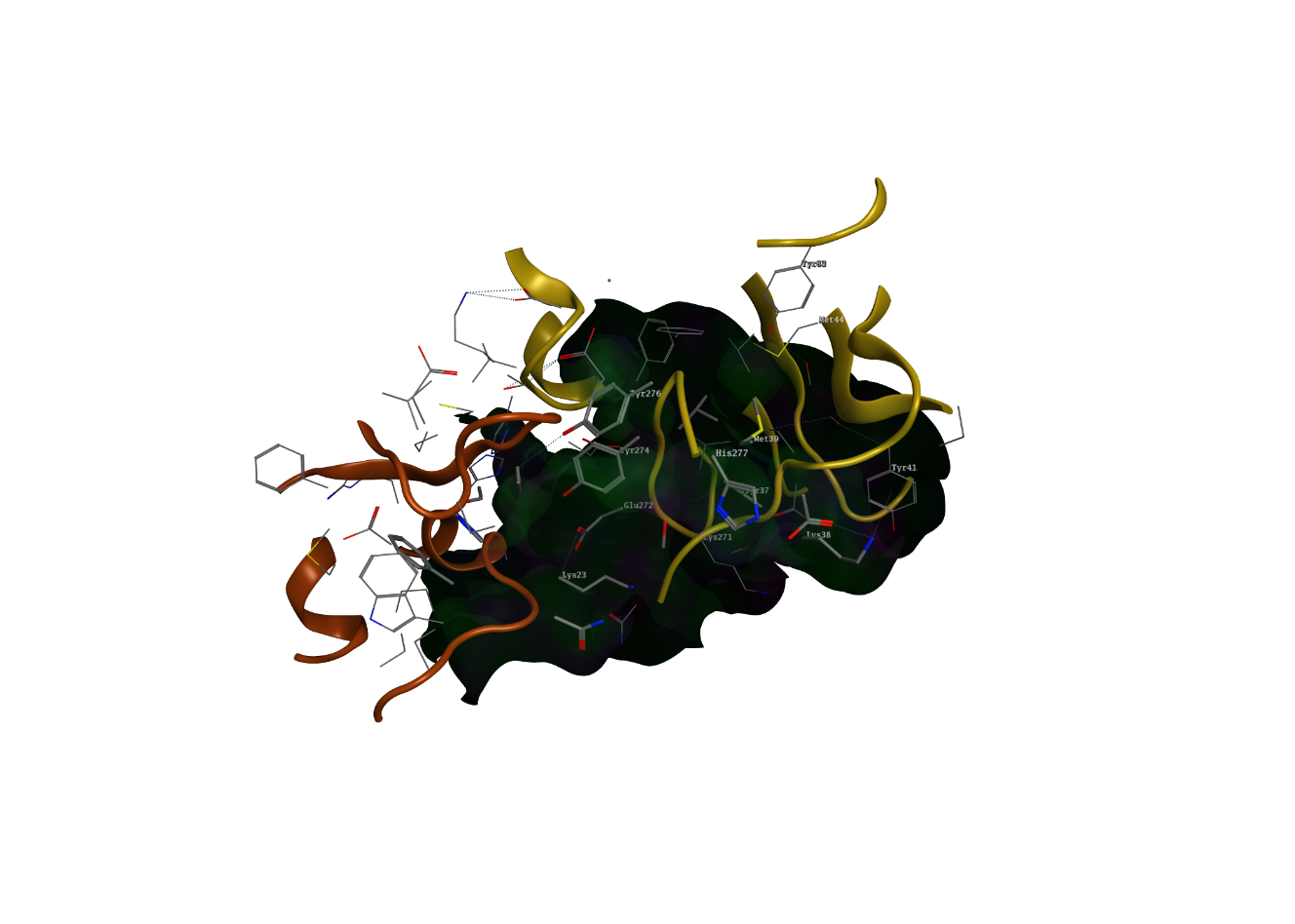

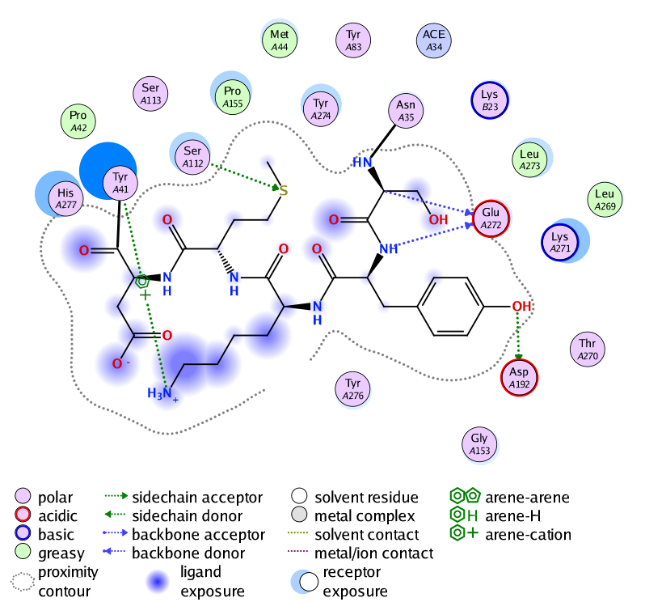


**S9B Fig. Protein-protein docking of Survivin and Caspase-9.** Protein-protein docking of survivin (brown) and caspase-9 (blue) was done by PatchDock Server and the result was refined by FireDock Server.


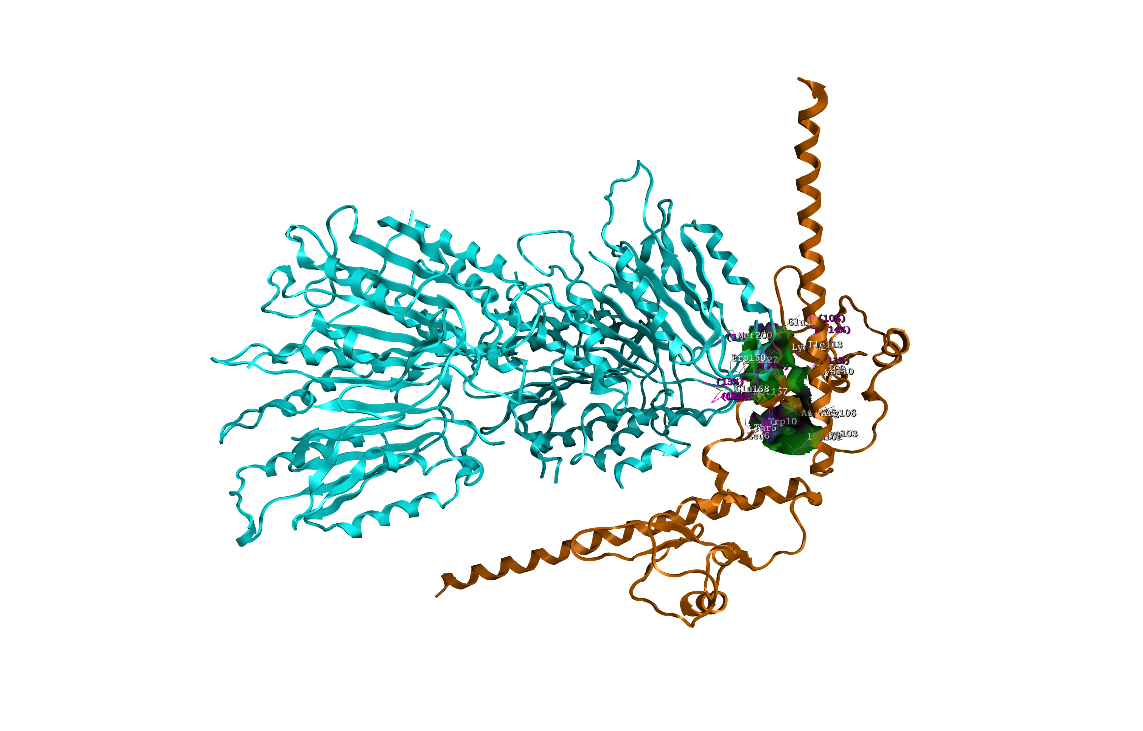

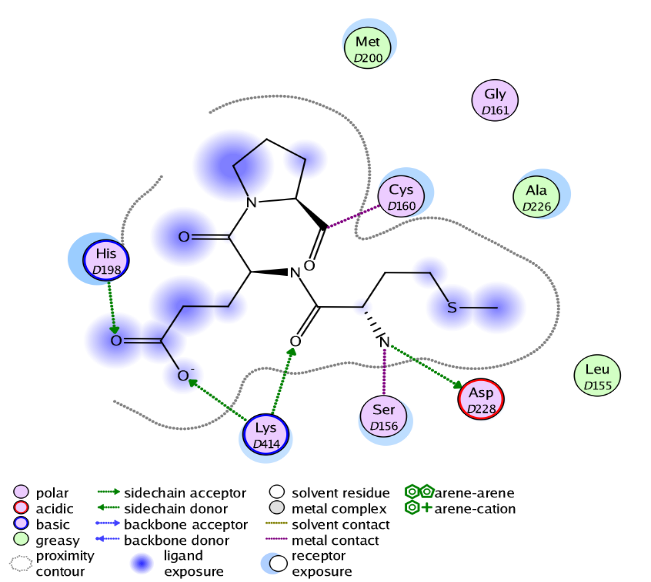

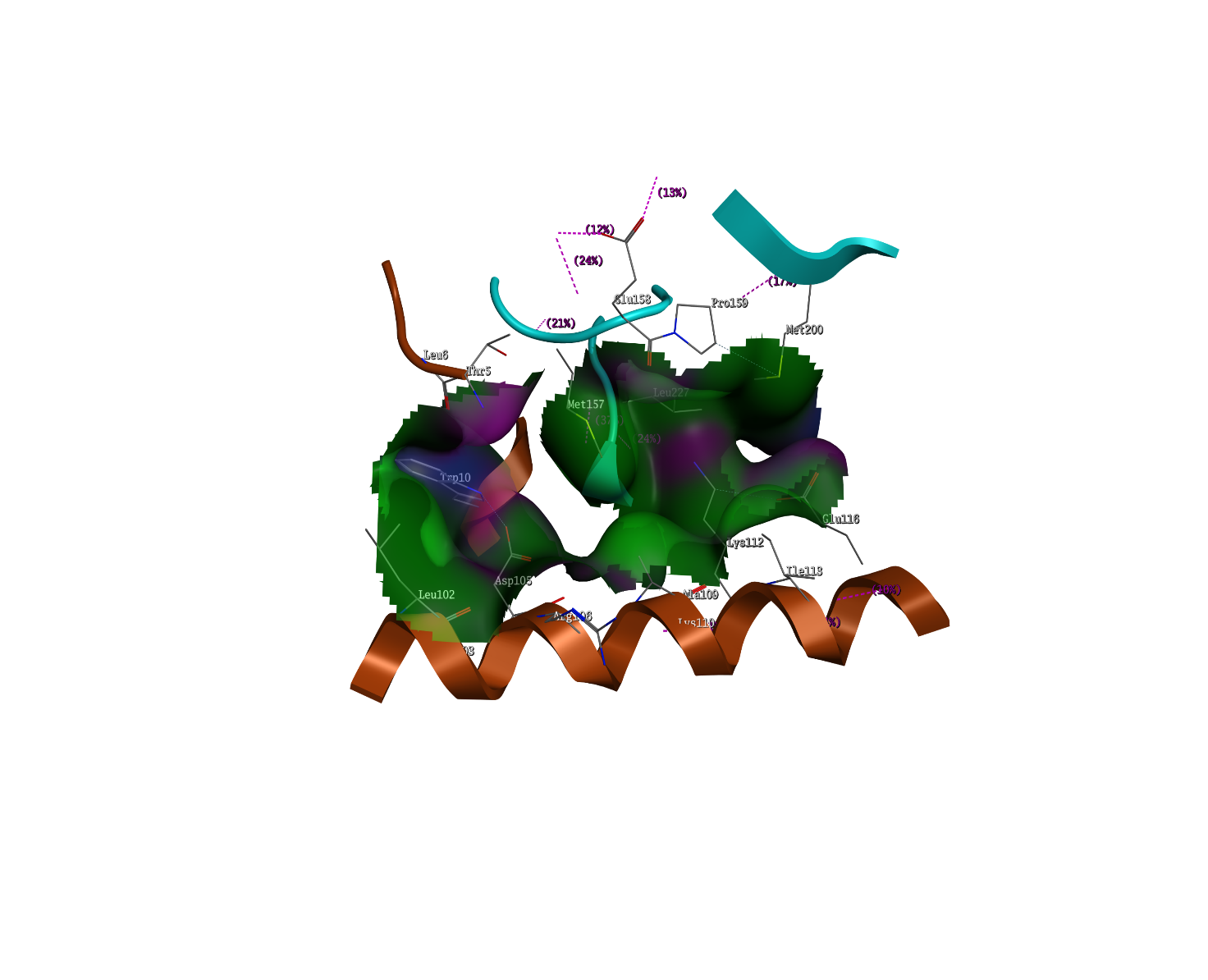


**S9C Fig. Protein – protein docking of Caspase-9 and Caspase-3.** Protein-protein docking of caspase-9 (blue) and caspase-3 (yellow) was done by PatchDock Server and the result was refined by FireDock Server.


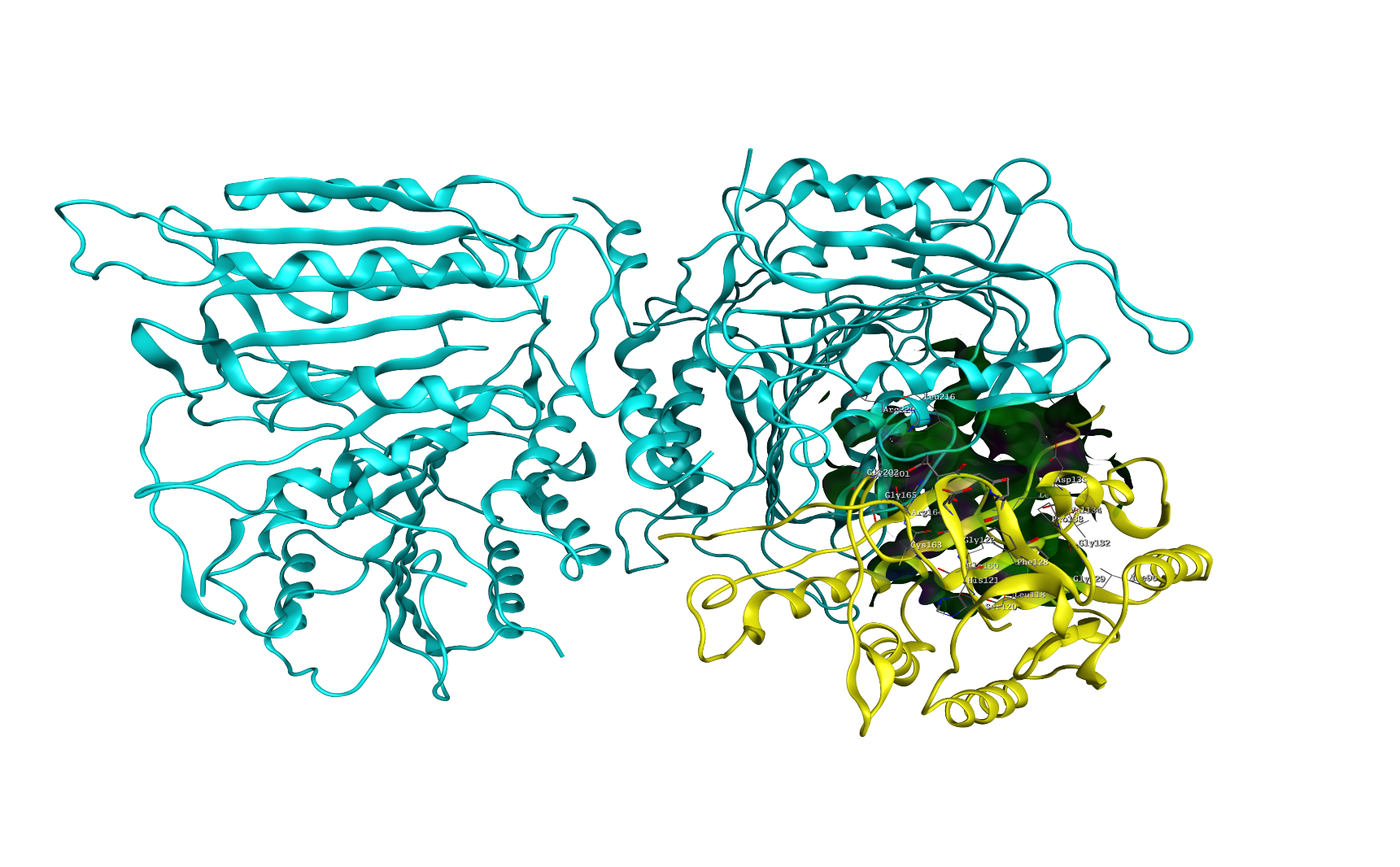

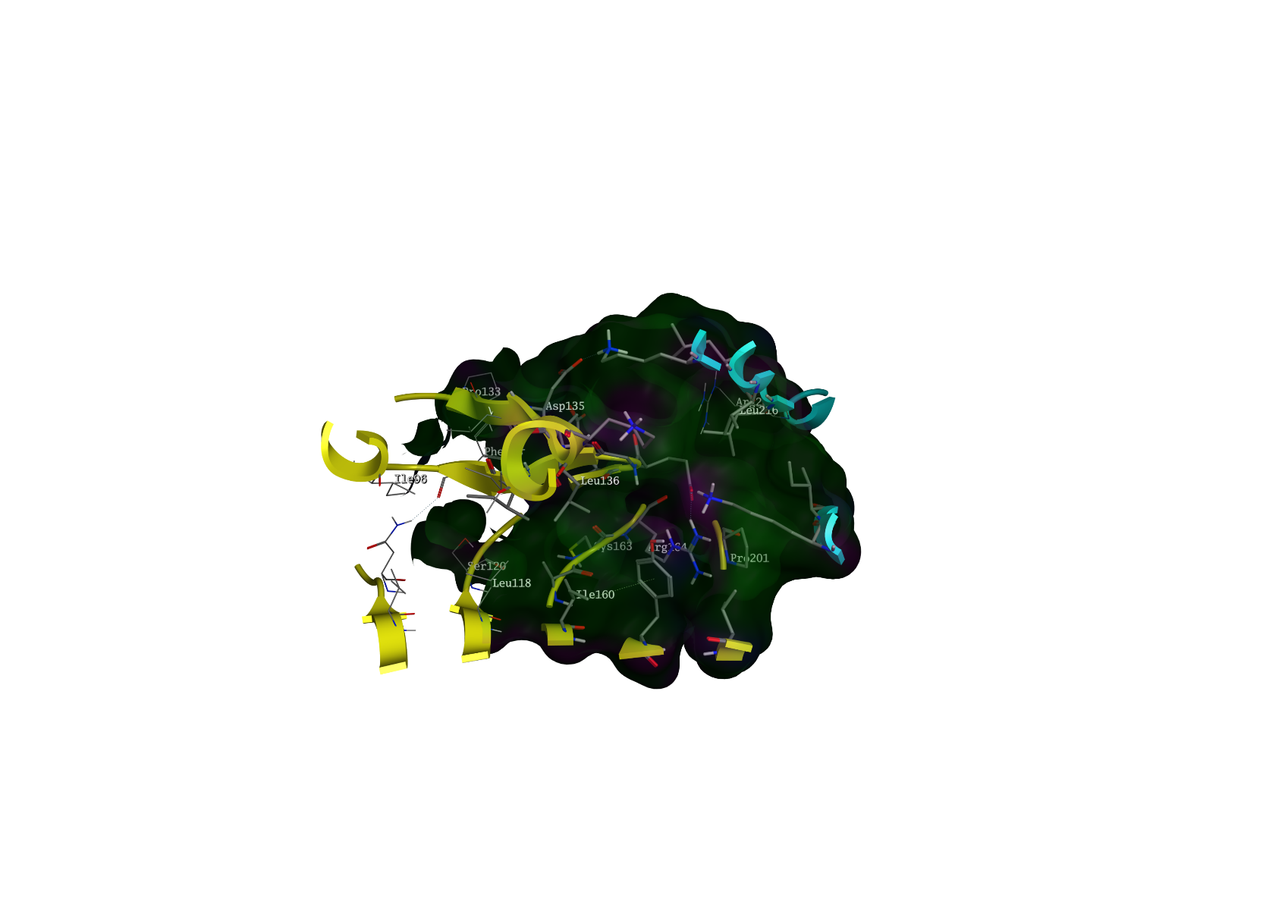

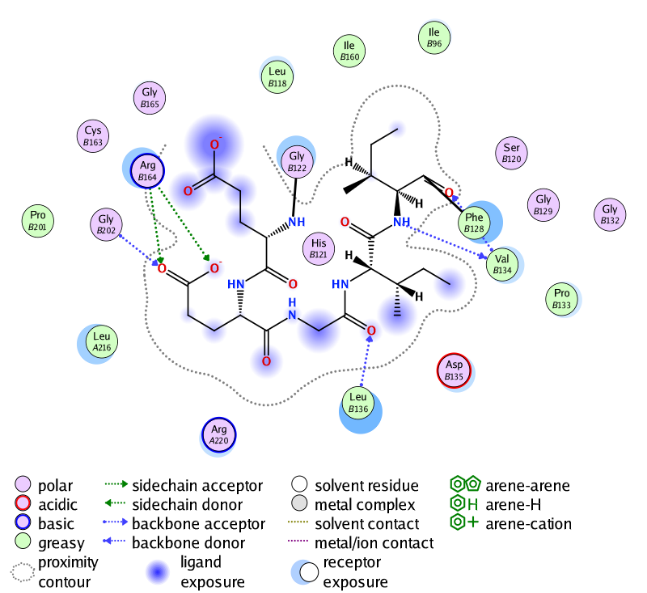


**S9D Fig. Molecular interaction of Survivin, Caspase-9 and andrographolide.** Molecular interaction of survivin (brown), caspase-9 (blue) and andrographolide (purple) was done by PatchDock Server and the result was refined by FireDock Server.


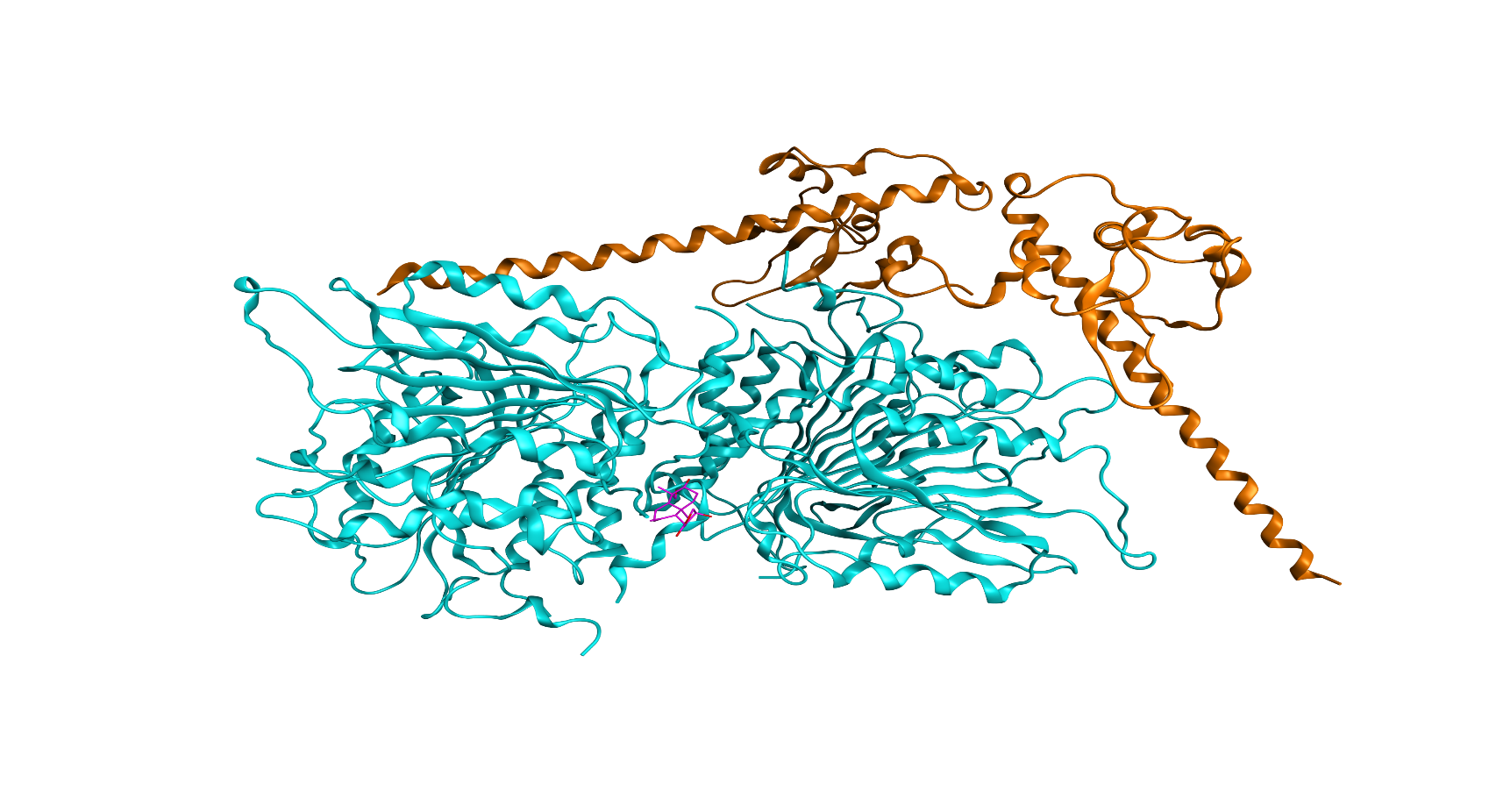

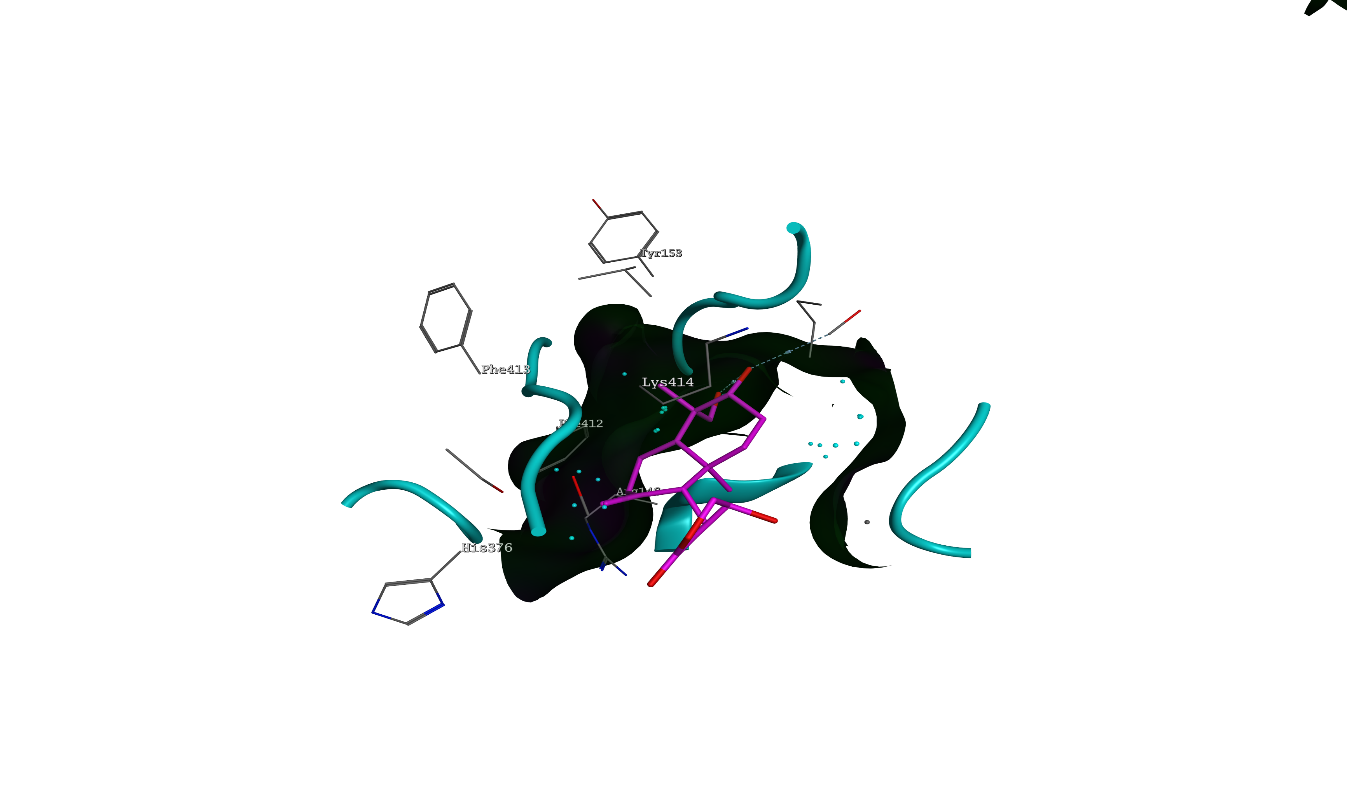

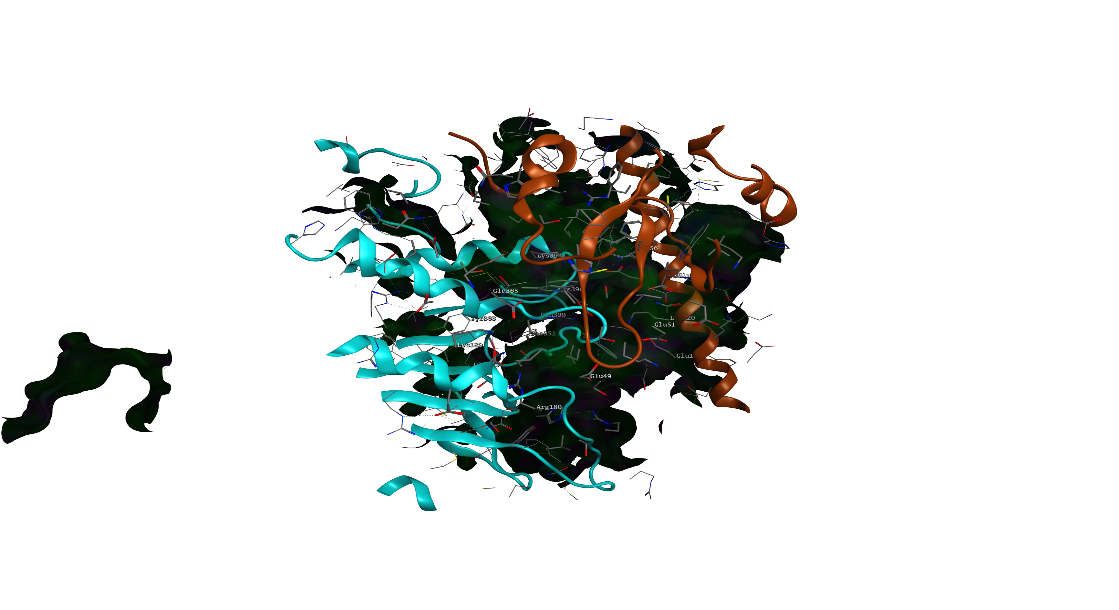

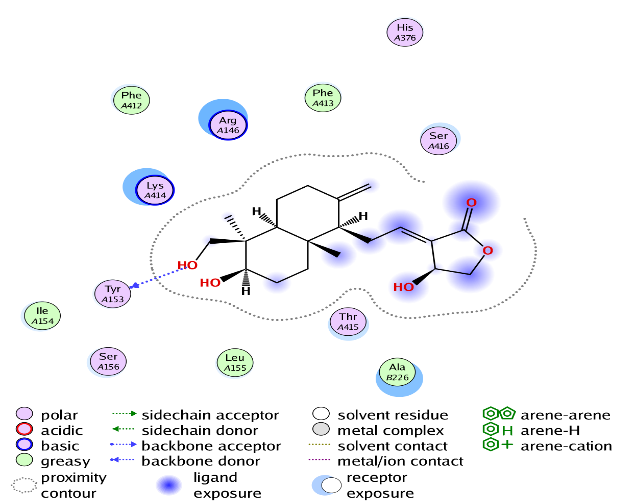

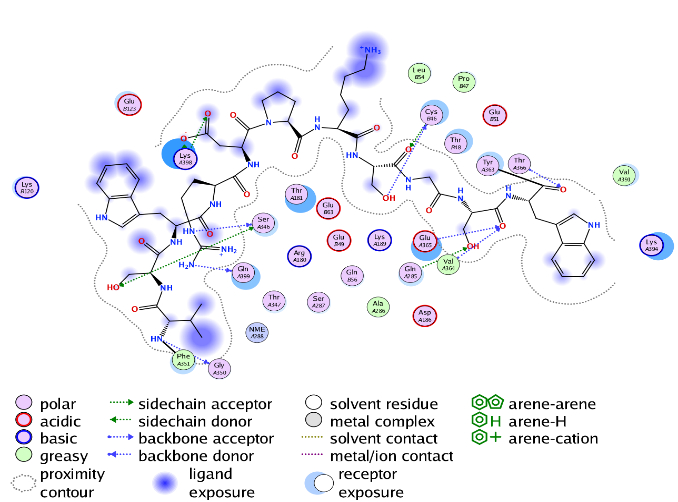


**S9E Fig. Molecular interaction of Caspase-3, Caspase-9 and andrographolide.** Molecular interaction of caspase-3 (yellow), caspase-9 (blue) and andrographolide (purple) was done by PatchDock Server and the result was refined by FireDock Server.


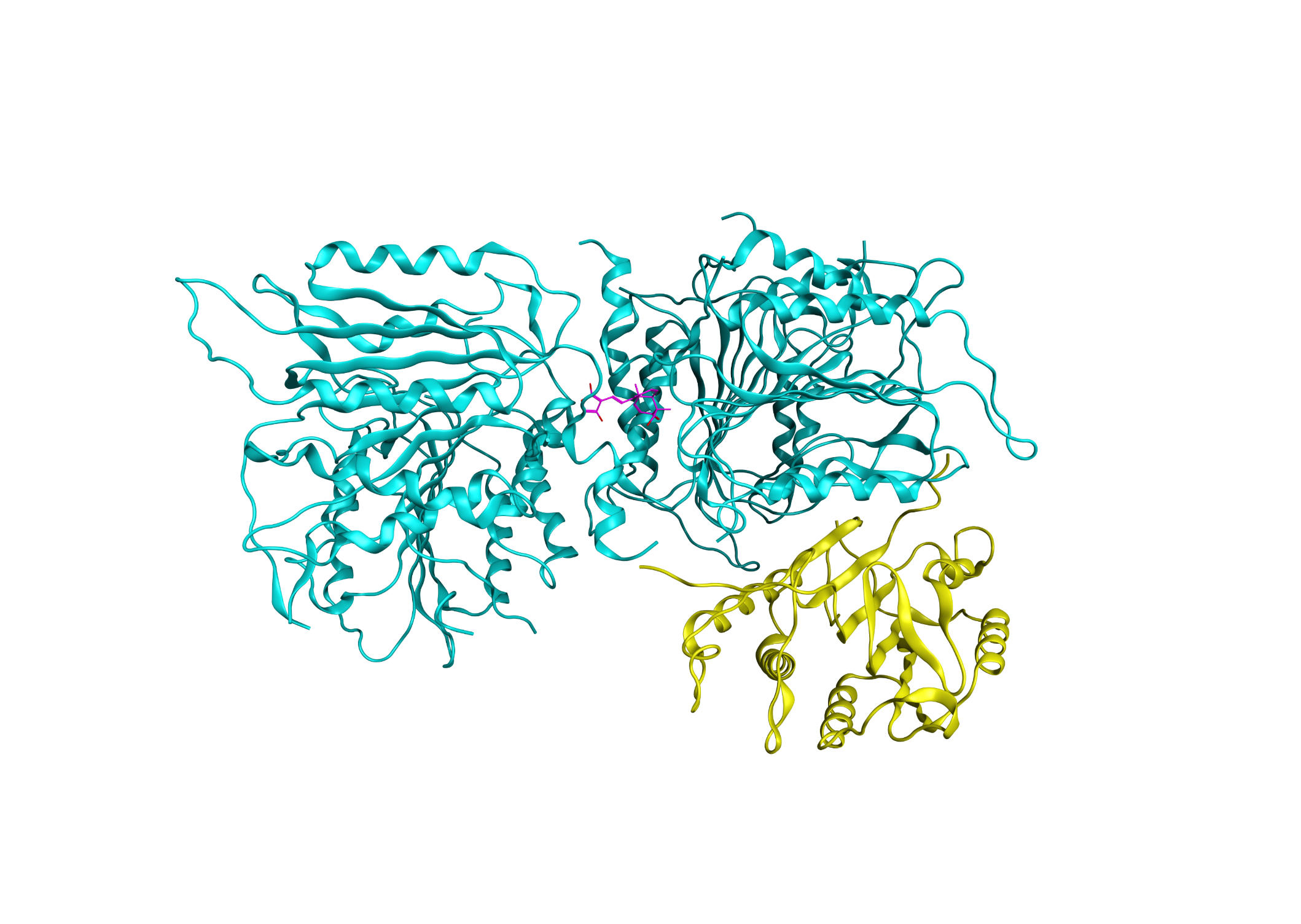

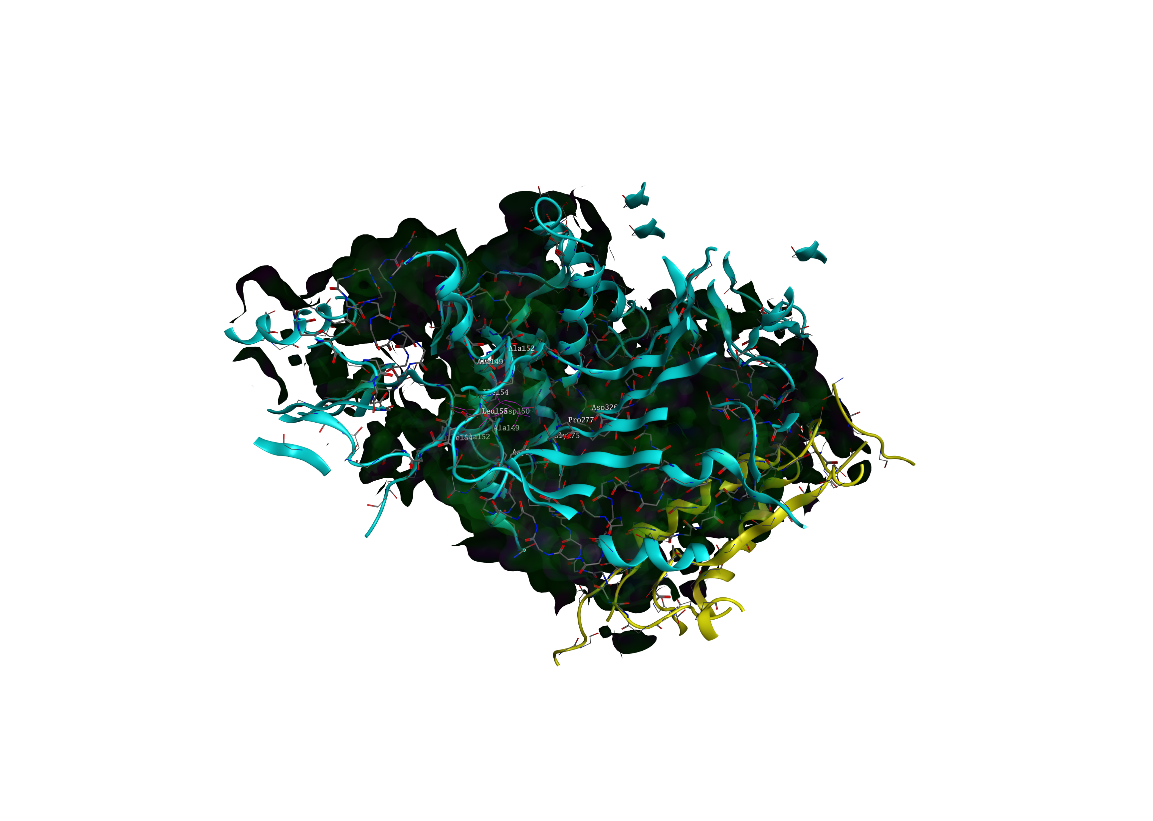

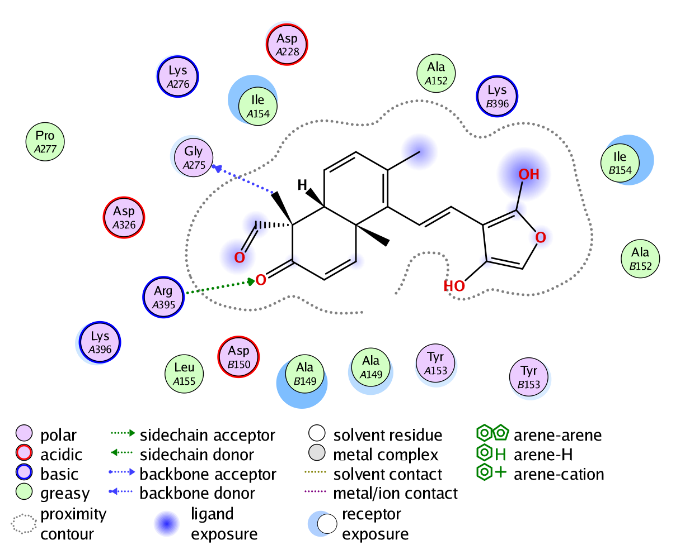

Supplement: S9 Fig — (DOCX) [file pone.0240020.s009.Docx]
