# Supplementary material for: In silico and in vitro studies on the anti-cancer activity of andrographolide targeting survivin in human breast cancer stem cells
Source: PLoS One. 2020 Nov 19;15(11):e0240020. doi: 10.1371/journal.pone.0240020 (PMC7676700; doi:10.1371/journal.pone.0240020)
Supplement: S1 Table — (DOCX) [file pone.0240020.s010.docx]

**S1A Table.** **Energy values obtained in molecular docking calculations with survivin**.

The blind docking process using Triangle Matcher Placement method with London dG scoring function and the refinement using Induced Fit method with GBVI/WSA dG scoring.^21^

| Mol | rseq | mseq | S | rmsd_refine | E_conf | E_place | E_score1 | E_refine | E_score2 |
| --- | --- | --- | --- | --- | --- | --- | --- | --- | --- |
| Andrographolide | 1 | 1 | -10.5464 | 0.839404 | 141.2636 | -69.9318 | -8.4905 | 26.0455 | -10.5644 |
| Curcumin | 1 | 2 | -9.0833 | 2.596608 | -42.8035 | -63.7575 | -9.7404 | -26.1663 | -9.0833 |
| 6-gingerol | 1 | 3 | -8.8067 | 1.790486 | -21.6985 | -50.7852 | -10.0325 | -19.1013 | -8.8067 |
| 8-gingerol | 1 | 4 | -9.9958 | 1.71679 | -25.2339 | -53.7944 | -8.4814 | -23.6568 | -9.9958 |
| 10-gingerol | 1 | 5 | -9.2800 | 1.344365 | -23.1035 | -60.171 | -8.6462 | -21.4217 | -9.2800 |
| Mangostin | 1 | 6 | -9.2930 | 2.273932 | -1.06746 | -71.4785 | -11.4785 | 13.8237 | -9.2930 |
| Rocaglamide | 1 | 7 | -14.2737 | 1.059464 | 2.7806 | -46.4227 | -10.09 | 8.7785 | -14.2737 |

**S1B Table. Energy values obtained in molecular docking calculations with survivin**.

The docking process on Thr34 site using Triangle Matcher Placement method with London dG scoring function and the refinement using Induced Fit method with GBVI/WSA dG scoring.^21^

| mol | rseq | mseq | S | rmsd_refine | E_conf | E_place | E_score1 | E_refine | E_score2 |
| --- | --- | --- | --- | --- | --- | --- | --- | --- | --- |
| Rocaglamide | 1 | 1 | -5.38513 | 2.97571 | 18.79905 | -62.5344 | -6.47324 | -26.3377 | -5.38513 |
| Andrographolide | 1 | 2 | -8.26169 | 2.2407 | 21.6985 | -40.5772 | -6.82423 | -3.72591 | -8.26169 |
