# Supplementary material for: In silico and in vitro studies on the anti-cancer activity of andrographolide targeting survivin in human breast cancer stem cells
Source: PLoS One. 2020 Nov 19;15(11):e0240020. doi: 10.1371/journal.pone.0240020 (PMC7676700; doi:10.1371/journal.pone.0240020)
Supplement: S2 Table — (DOCX) [file pone.0240020.s011.docx]

**S2A Table. BCSC viability after 24 hour andrographolide treatment**

| **Andrographolide** | | | | | | |
| --- | --- | --- | --- | --- | --- | --- |
| mM | 0.0000 | 0.0375 | 0.0750 | 0.1500 | 0.3000 | 0.6000 |
| Total | 345000 | 330000 | 345000 | 345000 | 315000 | 330000 |
| Live | 315000 | 295000 | 295000 | 275000 | 195000 | 29000 |
| Death | 30000 | 35000 | 50000 | 65000 | 115000 | 300000 |
| Viability | 91.3% | 89.3% | 85.5% | 79.7% | 61.9% | 8.7% |
| **DMSO** | | | | | | |
| mM | 0.0000 | 0.0375 | 0.0750 | 0.1500 | 0.3000 | 0.6000 |
| Total | 345000 | 315000 | 345000 | 325000 | 325000 | 315000 |
| Live | 315000 | 285000 | 305000 | 280000 | 270000 | 245000 |
| Death | 30000 | 30000 | 40000 | 45000 | 55000 | 65000 |
| Viability | 91.3% | 90.4% | 88.4% | 86.1% | 83.0% | 78.0% |

**S2B Table. MSC viability of after 24 hour andrographolide treatment.**

| **Andrographolide** | | | | | | |
| --- | --- | --- | --- | --- | --- | --- |
| mM | 0.0000 | 0.0375 | 0.0750 | 0.1500 | 0.3000 | 0.6000 |
| Total | 100000 | 100000 | 104000 | 99500 | 97000 | 105000 |
| Live | 94700 | 91400 | 92096 | 85950 | 81550 | 85800 |
| Death | 5300 | 8600 | 11904 | 13550 | 15450 | 14700 |
| Viability | 94.7% | 91.4% | 88.6% | 86.4% | 84.1% | 81.7% |
| **DMSO** | | | | | | |
| mM | 0.0000 | 0.0375 | 0.0750 | 0.1500 | 0.3000 | 0.6000 |
| Total | 100000 | 105000 | 98500 | 104500 | 104000 | 99000 |
| Live | 94700 | 96750 | 89400 | 92000 | 88750 | 81550 |
| Death | 5300 | 8250 | 9100 | 12500 | 15750 | 17450 |
| Viability | 94.7% | 92.2% | 90.8% | 87.8% | 85.3% | 82.4% |

**S2C Table. Viability of human non-BCSCs (CD24-/CD44- breast cancer cells and human MCF-7 breast cancer cell line) after 24 hour andrographolide treatment**

| **Treatment** | **Mean**  **Live Cells** | **Mean**  **% Viability** | **SD** |
| --- | --- | --- | --- |
| **CD24-/CD44-** **cells** | | | |
| DMSO | 263333.3 | 100.00 | 18.63 |
|  |  |  |  |
|  |  |  |  |
| Andro 0,075mM | 177833.3 | 67.53 | 4.06 |
|  |  |  |  |
|  |  |  |  |
| Andro 0,15mM | 110666.7 | 42.03 | 4.94 |
|  |  |  |  |
|  |  |  |  |
| Andro 0,3mM | 78666.67 | 29.87 | 6.29 |
|  |  |  |  |
|  |  |  |  |
| Andro 0,6mM | 780 | 0.30 | 0.51 |
|  |  |  |  |
|  |  |  |  |
| **MCF-7** **cells** | | | |
| DMSO | 138666.7 | 100.00 | 16.88 |
|  |  |  |  |
|  |  |  |  |
| Andro 0,075mM | 77833.33 | 56.13 | 4.18 |
|  |  |  |  |
|  |  |  |  |
| Andro 0,15mM | 41316.67 | 29.80 | 1.96 |
|  |  |  |  |
|  |  |  |  |
| Andro 0,3mM | 0 | 0.00 | 0.00 |
|  |  |  |  |
|  |  |  |  |
| Andro 0,6mM | 0 | 0.00 | 0.00 |
|  |  |  |  |
|  |  |  |  |
