# Supplementary material for: In silico and in vitro studies on the anti-cancer activity of andrographolide targeting survivin in human breast cancer stem cells
Source: PLoS One. 2020 Nov 19;15(11):e0240020. doi: 10.1371/journal.pone.0240020 (PMC7676700; doi:10.1371/journal.pone.0240020)
Supplement: S4 Table — (DOCX) [file pone.0240020.s013.docx]

**S4 Table. ELISA Data of Total and Phosphorylated Survivin protein levels in BCSCs treated with various andrographolide.**
