# Supplementary material for: In silico and in vitro studies on the anti-cancer activity of andrographolide targeting survivin in human breast cancer stem cells
Source: PLoS One. 2020 Nov 19;15(11):e0240020. doi: 10.1371/journal.pone.0240020 (PMC7676700; doi:10.1371/journal.pone.0240020)
Supplement: S5 Table — (DOCX) [file pone.0240020.s014.docx]

| Complex Receptor-Ligand | Binding Energy  (kcal/mol) | Amino acid interactions | Hydrogen Bonds |
| --- | --- | --- | --- |
| Survivin-Smac | -6.65 | Leu54; Glu63; Glu65; Glu76; His80; Asp71 | Leu64  Lys62 |
| Survivin-Smac-Andro | -3.61 | Val2; Ile4; Gly66 | Pro3  Glu65 |
| Survivin-Andro-Smac | -2.67 | Lys62; Glu65; Gly66; Lys79; Glu76; Asp71 |  |
| Smac-Andro | -3.06 | Ala1; Val2; Ile4 | Pro3 |

**S5 Table. Binding energy of survivin and Smac/DIABLO in the presence of andrographolide**

Molecular docking was performed using autodock software version 4.2 (see also S8 Figure). The result demonstrated that the binding energy of survivin and Smac/DIABLO was increased in the presence of andrographolide, indicating the decrease of binding affinity between survivin and Smac/DIABLO.
