# Supplementary material for: In silico and in vitro studies on the anti-cancer activity of andrographolide targeting survivin in human breast cancer stem cells
Source: PLoS One. 2020 Nov 19;15(11):e0240020. doi: 10.1371/journal.pone.0240020 (PMC7676700; doi:10.1371/journal.pone.0240020)
Supplement: Attachment — Submitted filename: revision plos one.docx [file pone.0240020.s015.docx]

1. The authors have studied the expression levels of caspase 3, caspase 9 and survivin at the mRNA level. After translation in the cytoplasm ,the proteins undergo a lot of post translational modifications. So, my suggestion would be to please check the expression levels of caspase 9, caspase 3 and survivin at the protein levels using standard western blot or flow cytometry protocols.
2. The authors have selected 7 natural compounds from medicinal plants, curcumin, rocaglamide, α-mangostin, 6-gingerol, 8-gingerol, 10-gingerol, and Andrographolide, all of which have anticancer properties. However the authors did not mention why they selected these specific compounds, given the fact that there are a lot of other available natural compounds, having anticancer potential.
3. Apoptosis is a programmed cell death process involving the participation of lot of proteins. Survivin functions by binding to pro-apoptotic proteins SMAC/DIABLO, which in turn prevents caspase activation. So, studying the expression levels of these proteins at the genetic and protein levels are also required.
4. In the graphical abstract part replace the second caspase 9 with caspase 3.
5. The effect of Andrographolide on breast cancer stem cells is quite remarkable but IC50 value of 320μM is pretty high dose on cancer cells, considering the fact that marketed drugs against breast cancer are effective at 10-20μM concentration. What could be the other possible reasons of selecting such a compound for anticancer activity, other than the fact that its non toxic to normal stem cells?
6. In the legend fig 5B ‘BCSC morphology treated with 0.06 mM andrographolide’, recheck if it is 0.06mM or 0.6mM?
7. Include the statistical significances in fig.7B
