# Supplementary material for: In silico and in vitro studies on the anti-cancer activity of andrographolide targeting survivin in human breast cancer stem cells
Source: PLoS One. 2020 Nov 19;15(11):e0240020. doi: 10.1371/journal.pone.0240020 (PMC7676700; doi:10.1371/journal.pone.0240020)
Supplement: Attachment — Submitted filename: Response to Reviewers (rev).docx [file pone.0240020.s016.docx]

**PONE-D-20-03995
In silico and in vitro studies on the anti-cancer activity of andrographolide targeting survivin in human breast cancer stem cells**

Jakarta, July 30th, 2020

Dear Arun Rishi, Ph.D.
Academic Editor
PLOS ONE

First of all, I would like to extend our sincere thanks to the PLOS ONE academic editor and reviewers for the fruitful comments and also for giving us the opportunity to revise our manuscript in this most difficult time due to pandemic situation. Herewith, I kindly send you the responds to each point raised by the academic editor and reviewers in the following Tables.

**Responses to Academic Editor’s comments**

| **No.** | **Reviewer’s comments** | **Responses** | **Manuscript Revision** |
| --- | --- | --- | --- |
| 1. | Please provide additional details regarding consent sought from patients to obtain the primary breast cancer stem cells and mesenchymal stem cells used in this study. | We have provided the following details:  “Breast cancer tissue has been collected from a triple negative breast cancer patient underwent surgery. Written informed consent form has been signed by the patient prior to collecting the specimen. Mesenchymal stem cells (MSCs) were obtained from Prof. Endang Winiati Bachtiar (Laboratory of Oral Biology Faculty of Dentistry, Universitas Indonesia) who has isolated the MSCs from dental pulp of a patient underwent tooth extraction with a written informed consent signed by the patient and approved by the Ethics Commission of Research and Community Service Institution at Bogor Agricultural Institute (No. 05-2015 IPB). This study was conducted according to the ethical principles.” | Page 5-6  Line 136-144 |
| 2. | In the ethics statement in the Methods and online submission information, please ensure that you have specified (a) whether consent was informed and (b) what type you obtained (for instance, written or verbal, and if verbal, how it was documented and witnessed). |  |  |
| 3. | On the contrary, if these cells are commercially available cell lines, please include this information and state the specific name of the cell lines. | In this study, we did not use any commercially available cell lines | - |
| 4. | Please provide additional information about each of the cells used in this work, including any quality control testing procedures (mycoplasma testing). For more information, please see <http://journals.plos.org/plosone/s/submission-guidelines#loc-cell-lines>. | Both BCSCs and MSCs used in this study are established cells that have been utilized in the previous studies (References has been added). The mycoplasma testing resulted in a negative compared to the positive control.  The following information has been added in the manuscript:  “Human BCSCs (CD24-/CD44+) have been isolated from the primary culture of breast cancer cells (Granted patent from the General Directorate of Intellectual Property Right, Ministry of Law and Human Right, Republic of Indonesia No. IDP00056854) and characterized for their pluripotency and tumorigenicity (Granted patent from the General Directorate of Intellectual Property Right, Ministry of Law and Human Right, Republic of Indonesia No. IDP000060309). The human BCSCs and MSCs have been used in the previous studies [33-34].”  “The MSCs has been utilized in the previous study [33].” | Page 6  Line 147-152.  Page 6  Line 143 |
| 5. | To comply with PLOS ONE submission guidelines, in your Methods section, please provide additional information regarding your statistical analyses. For more information on PLOS ONE's expectations for statistical reporting, please see <https://journals.plos.org/plosone/s/submission-guidelines.#loc-statistical-reporting>. | We have added the following information:  **Statistical analysis**  “Data were analyzed using SPSS software version 26. Data is presented as mean ± standard deviation (SD) of three independent experiments with three replicates. Coefficient of variation (COV) is calculated as a percentage ratio of the SD to the mean of expression level. The COV value expresses the homogeneity of data distribution. Normality tests performed on all experiment data were Kolmogorof-Smirnov test and the Levene homogeneity test. Since the data distribution is normal and homogenous, the analysis was continued with the parametric test of one-way analysis of unpaired variance (one-way ANOVA, unrelated) followed by the Tuckey test to show the differences between groups”. | Page 9  Line 226-234 |
| 6. | Thank you for including the following funding information in your acknowlegdements section; "This work was funded by the PDUPT Grant (NKB-1564/UN2.R3.1/HKP.05.00/2019) from the Ministry of Research, Technology and Higher Education of the Republic of Indonesia. The funders had no role in study design, data collection and analysis, decision to publish, or preparation of the manuscript. "  We note that you have provided funding information that is not currently declared in your Funding Statement. However, funding information should not appear in the Acknowledgments section or other areas of your manuscript. We will only publish funding information present in the Funding Statement section of the online submission form.  Please remove any funding-related text from the manuscript and let us know how you would like to update your Funding Statement. Currently, your Funding Statement reads as follows:  "The funders had no role in study design, data collection and analysis, decision to publish, or preparation of the manuscript." | Thank you for correcting this mistake. We removed any funding-related text from the manuscript.  Please update our Funding Statement as follows:  "This work was funded by the PDUPT Grant (NKB-1564/UN2.R3.1/HKP.05.00/2019) from the Ministry of Research, Technology and Higher Education of the Republic of Indonesia. The funders had no role in study design, data collection and analysis, decision to publish, or preparation of the manuscript." |  |
| 7. | In your revised cover letter, please address the following prompts:  If there are no restrictions, please upload the minimal anonymized data set necessary to replicate your study findings as either Supporting Information files or to a stable, public repository and provide us with the relevant URLs, DOIs, or accession numbers.  For a list of acceptable repositories, please see <http://journals.plos.org/plosone/s/data-availability#loc-recommended-repositories>.  We will update your Data Availability statement on your behalf to reflect the information you provide. | The minimal anonymized data set necessary to replicate our study findings can be found in Supporting Information files.  Please update our data availability as follows:  “All relevant data are within the manuscript and its Supporting Information files.” | Page 21  Line 519 |
| 8. | In the Methods section, please provide the primer sequences and source of the primers for the 18srRNA mRNA amplifications. | We have provided the sources of all primers used in this study, as follows:  “The primers for survivin, caspase-9, caspase-3, and 18S rRNA cDNA amplification were designed using the Primer-Blast program and NCBI Gene Bank [NM_001168.2, NM_01278054.1, NM_004346.4, and NC_000021.9, respectively].” | Page 7  Line 182-184 |
| 9. | In the Methods section, please provide the product number and lot number of the Andrographolide purchased from Sigma-Aldrich for this study. | We have provided the following information for andrographolide:  “Product No. 365645; Lot No. MKBN8939V” | Page 6  Line 161 |

**Responses to Reviewer I’s comments**

| **No.** | **Reviewer’s comments** | **Responses** | **Manuscript Revision** |
| --- | --- | --- | --- |
| 1. | Fig. 4C is repeated | We have deleted the Fig. 4C | Page 14-15  Line 326-333 |
| 2. | The authors are recommended to investigate the anti-cancer capacity of andrographolide and survivin inhibition by andrographolide in other non-BCSCs. It is better to compare the anti-cancer activity of andrographolide in BCSCs and non-BCSCs. | We have compared the anti-cancer activity of andrographolide in BCSCs and non-BCSCS. The following statement has been added in the manuscript.  “We indicated that the CC_50_ value of andrographolide in BCSCs is significantly higher than that in non-BCSCs such as human CD24-/CD44- breast cancer cells and human MCF-7 breast cancer cells (S2 Figure).” | Page 18  Line 420-422 |
| 3. | For Fig.5B, please clearly label the drug concentration used in the study. | Thank you for your correction.  We have revised the drug concentration.  In Figure 4D we have added:  “BCSC morphology treated with 0.6 mM andrographolide”. | Page 15  Line 333 |
| 4. | In the legend of Fig.5B (“BCSC morphology treated with 0.06 mM andrographolide”). Please make sure the drug concentration is corrected. |  |  |
| 5. | Fig.4 and Fig.5 should be merged into one Figure. | We have merged Fig. 4 and Fig. 5 into one Figure. | Page 14-15  Line 326-333 |
| 6. | For Fig.6, the authors are strongly recommended to detect survivin and phosphorylated survivin protein level by using immunoblotting assay. | We have detected the protein level of survivin and phosphorylated survivin using immunoblotting assay.  The data regarding this immunoblotting assay has been added in the manuscript. | Page 8-9  Line 204-214  Page 15-16  Line 356-360  Page 16  Line 367-368  Page 19  Line 443-445 |
| 7. | For Fig.7, the authors need to further measure the activation of apoptosis-associated proteins (e.g. PARP, caspase-3, caspase-9, and so on) by immunoblotting. | We have performed the immunoblotting assay to demonstrate the activation of caspase-9 and caspase-3.  The data regarding this immunoblotting assay has been added in the manuscript. | Page 8-9  Line 204-214  Page 16  Line 360-361  Page 16  Line 367-368  Page 20  Line 483-484  Line 487-490 |
| 8. | To make sure the determinant role of survivin inhibition by andrographolide in its anti-cancer activity in breast cancer stem cells, the authors need to show the proliferation rates of BCSCs and surviving-KO/KD BCSCs in their system. | We have performed the experiments to show the proliferation rates of BCSCs and survivin-inhibited BCSCs (using YM155 as a survivin inhibitor), as indicated by their doubling times.  The following result has been added in the manuscript:  “Our supporting data also revealed that the proliferation rate of BCSCs was inhibited when treated with 100 nM YM155, a survivin inhibitor [S3 Figure]. This suggests that the inhibition of survivin has a determinant role in the anti-cancer activity of andrographolide.” | Page 18  Line 430-432 |
| 9. | The authors need to clarify the statistical analysis in the Materials and Methods section. | We have added the following information:  **Statistical analysis**  “Data were analyzed using SPSS software version 26. Data is presented as mean ± standard deviation (SD) of three independent experiments with three replicates. Coefficient of variation (COV) is calculated as a percentage ratio of the SD to the mean of expression level. The COV value expresses the homogeneity of data distribution. Normality tests performed on all experiment data were Kolmogorof-Smirnov test and the Levene homogeneity test. Since the data distribution is normal and homogenous, the analysis was continued with the parametric test of one-way analysis of unpaired variance (one-way ANOVA, unrelated) followed by the Tuckey test to show the differences between groups.” | Page 9  Line 226-234 |

**Responses to Reviewer II’s comments**

| **No.** | **Reviewer’s comments** | **Responses** | **Manuscript Revision** |
| --- | --- | --- | --- |
| 1 | The authors have studied the expression levels of caspase 3, caspase 9 and survivin at the mRNA level. After translation in the cytoplasm ,the proteins undergo a lot of post translational modifications. So, my suggestion would be to please check the expression levels of caspase 9, caspase 3 and survivin at the protein levels using standard western blot or flow cytometry protocols. | We have performed the immunoblotting assay to demonstrate the activation of caspase-9 and caspase-3.  The data regarding this immunoblotting assay has been added in the manuscript. | Page 8-9  Line 204-214  Page 15-16  Line 356-361  Page 16  Line 367-368  Page 19  Line 443-445  Page 20  Line 483-484  Line 487-490 |
| 2 | The authors have selected 7 natural compounds from medicinal plants, curcumin, rocaglamide, α-mangostin, 6-gingerol, 8-gingerol, 10-gingerol, and Andrographolide, all of which have anticancer properties. However the authors did not mention why they selected these specific compounds, given the fact that there are a lot of other available natural compounds, having anticancer potential. | We have added the following informations:  “These selected active compounds are found among medicinal plants such as *Curcuma domestica*, *Curcuma xantorrhiza*, *Aglaia sp*., *Andrographis paniculata* and *Garcinia mangostana* that are endemic in Southeast Asian countries, including Indonesia [9-11]. Therefore, utilization of these compounds for anticancer offers a potential benefit, both economically and clinically.” | Page 3  Line 76-80 |
| 3 | Apoptosis is a programmed cell death process involving the participation of lot of proteins. Survivin functions by binding to pro-apoptotic proteins SMAC/DIABLO, which in turn prevents caspase activation. So, studying the expression levels of these proteins at the genetic and protein levels are also required. | The following information have been added in the manuscript:  “Another anti-apoptotic mechanism of survivin that should be considered is through its direct interaction with Smac/DIABLO in the cytosol during interphase [44]. Smac/DIABLO is a mitochondrial protein that potentiates apoptosis by activating caspase in the cytochrome c/Apaf-1/caspase-9 pathway [45]. In our *in silico* study, we confirm that survivin directly binds to Smac/DIABLO (S8 Figure) which in turn may prevent caspase activation and inhibit apoptosis [44]. Moreover, the presence of andrographolide decreased the binding affinity of survivin and Smac/DIABLO *in silico* (S5 Table) and reduced survivin level *in vitro*, allowing Smac/DIABLO to promote caspase-activated apoptosis, as shown by the Annexin V-FITC result (Figure 6). However, the effect of andrographolide treatment on caspase activation by Smac/DIABLO should be further elucidated.” | Page 19  Line 465-474 |
| 4 | In the graphical abstract part replace the second caspase 9 with caspase 3. | Thank you for your correction. We have replaced the second caspase-9 with caspase-3 in Figure 7. | Figure 7 |
| 5 | The effect of Andrographolide on breast cancer stem cells is quite remarkable but IC50 value of 320μM is pretty high dose on cancer cells, considering the fact that marketed drugs against breast cancer are effective at 10-20μM concentration. What could be the other possible reasons of selecting such a compound for anticancer activity, other than the fact that its non toxic to normal stem cells? | We agree with the Reviewer’s comment regarding the high CC50 value of BCSCs.  We have added the following information to respond to this comment.  “Although the effect of andrographolide on the BCSCs is quite remarkable, its CC_50_ value of 0.32 mM in the CD24-/CD44+ BCSCs is higher than the doses of most marketed drugs against breast cancer that are effective at a lower concentration. Nevertheless, the most anti-cancer that are effective at a lower concentration are targeting the major population of breast cancer cells without considering the presence of BCSCs. In this study, we specifically targeted the side population of breast cancer cells – BCSCs – that are known to be less sensitive to anti-cancer than the non-BCSCs [36-37]. We indicated that the CC_50_ value of andrographolide in BCSCs is significantly higher than that in non-BCSCs such as human CD24-/CD44- breast cancer cells and human MCF-7 breast cancer cells (S2 Figure). Hence, the BCSCs need higher doses of anti-cancer than the major part of breast cancer cells which are not stem cells. Despite the high dose of andrographolide needed to target survivin in BCSCs, the present data showed that this dose was still non-toxic to the normal stem cells (Figure 4). We found that andrographolide is not toxic to MSCs derived from dental pulp even at CC_90_ (0.6 mM) of andrographolide in human BCSCs.” | Page 18  Line 414-427 |
| 6 | In the legend fig 5B ‘BCSC morphology treated with 0.06 mM andrographolide’, recheck if it is 0.06mM or 0.6mM? | Thank you for your correction.  We have revised the drug concentration.  In Figure 4D we have added:  “BCSC morphology treated with 0.6 mM andrographolide”. | Page 15  Line 333 |
| 7 | Include the statistical significances in fig.7B | Thank you for your suggestion.  For apoptosis assay using flow cytometry, we pooled triplicates of each sample to obtain enough cells after andrographolide treatment. Andrographolide remarkably reduced viable cell number (up to ca. 90% with 0.6 mM andrographolide; Fig. 4A). Therefore, we could not determine the statistical significances in the apoptosis result. |  |

Thank you for considering this manuscript to be published in your highly reputable journal.

Sincerely,

**Prof. Dr.rer.physiol. dr. Septelia Inawati Wanandi**

Department of Biochemistry and Molecular Biology,

Faculty of Medicine, Universitas Indonesia, Indonesia

Email: [septelia.inawati@ui.ac.id](mailto:septelia.inawati@ui.ac.id); [septelia.inawati@gmail.com](mailto:septelia.inawati@gmail.com)

Phone: +6221-3910734
